# Intra- and inter-protein couplings of backbone motions underlie protein thiol-disulfide exchange cascade

**DOI:** 10.1038/s41598-018-33766-4

**Published:** 2018-10-18

**Authors:** Wenbo Zhang, Xiaogang Niu, Jienv Ding, Yunfei Hu, Changwen Jin

**Affiliations:** 1 College of Life Sciences, Peking University, Beijing, 100871 China; 2 College of Chemistry and Molecular Engineering, Peking University, Beijing, 100871 China; 3 Beijing Nuclear Magnetic Resonance Center, Peking University, Beijing, 100871 China; 4 Beijing National Laboratory for Molecular Sciences, Peking University, Beijing, 100871 China; 5 Present Address: National Institutes of Health, DHHS 1050 Boyles Street, Frederick, MD 21702 USA; 6 Present Address: Medical College of Soochow University, Suzhou, 215123 China

## Abstract

The thioredoxin (Trx)-coupled arsenate reductase (ArsC) is a family of enzymes that catalyzes the reduction of arsenate to arsenite in the arsenic detoxification pathway. The catalytic cycle involves a series of relayed intramolecular and intermolecular thiol-disulfide exchange reactions. Structures at different reaction stages have been determined, suggesting significant conformational fluctuations along the reaction pathway. Herein, we use two state-of-the-art NMR methods, the chemical exchange saturation transfer (CEST) and the CPMG-based relaxation dispersion (CPMG RD) experiments, to probe the conformational dynamics of *B. subtilis* ArsC in all reaction stages, namely the enzymatic active reduced state, the intra-molecular C10–C82 disulfide-bonded intermediate state, the inactive oxidized state, and the inter-molecular disulfide-bonded protein complex with Trx. Our results reveal highly rugged energy landscapes in the active reduced state, and suggest global collective motions in both the C10–C82 disulfide-bonded intermediate and the mixed-disulfide Trx-ArsC complex.

## Introduction

Protein thiol-disulfide exchange reactions play fundamental roles in living systems, represented by the thioredoxin (Trx) and glutaredoxin (Grx) systems that maintain the cytoplasmic reducing environment, the protein DsbA that catalyzes the formation of protein disulfide bonds in bacterial periplasm, as well as the protein disulfide isomerase (PDI) proteins that facilitate correct disulfide bonding^1–5^. Although extensive studies have been carried out to elucidate the underlying mechanism for protein thiol-disulfide exchange reactions^1–3,6–10^, there still remain much controversies as well as challenges, particularly in the case of inter-protein disulfide exchanges involving transiently formed protein-protein mixed disulfide complexes^3,4^. The Trx-coupled arsenate reductase (ArsC) in Gram-positive bacteria like *Bacillus subtilis* and *Staphylococcus aureus* catalyzes the reduction of arsenate to arsenite as part of the arsenic detoxification pathway, and the oxidized inactive form of ArsC is reactivated by the upstream Trx system^11–16^. The enzymatic reaction of arsenate reduction occurs through an intramolecular cascade of thiol-disulfide exchanges involving three catalytic active cysteines in ArsC, whereas the regeneration of ArsC activity is accomplished by an inter-protein thiol-disulfide exchange with Trx. The involvement of both intra- and inter-protein thiol-disulfide exchange reactions in a single catalytic cycle makes the Trx-coupled ArsC an attractive model system for investigating protein thiol-disulfide exchange mechanisms. Moreover, the high-resolution NMR structure of *B. subtilis* Trx-ArsC mixed-disulfide intermediate^17^ is one of the few protein-protein complex structures of Trx with a downstream protein determined thus far, providing the structural basis for investigating the inter-protein disulfide exchanges.

**Figure 1:**
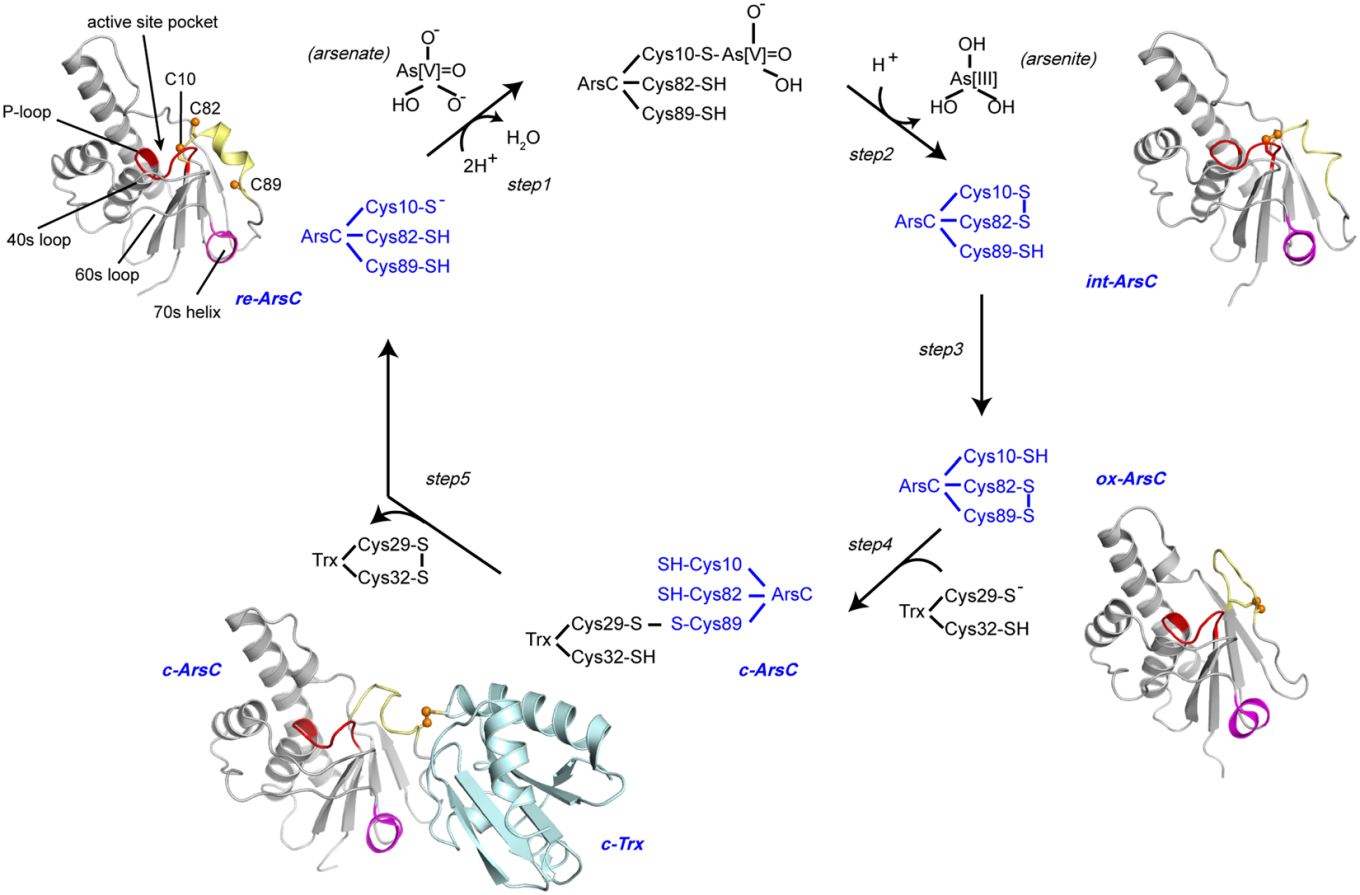
The catalytic mechanism of *B. subtilis* ArsC. Schematic presentation of the catalytic and re-activation cycle of *B. subtilis* ArsC. The four essential redox states *re-ArsC*, *int-ArsC*, *ox-ArsC* and *c-ArsC* are colored in blue. The structures of *re-ArsC*, *ox-ArsC* and *c-ArsC* (PDB entries 1z2d, 1z2e, and 2ipa) and a structure model of the C10–C82 disulfide-bonded *int-ArsC* generated using AMBER^54^ are shown. The active site P-loop, the three active cysteine residues and other important structural regions are shown in the structure of *re-ArsC*.

It is becoming commonly accepted that fluctuations of the protein conformational dynamics play a critical role in driving the enzymatic catalysis reactions. For thiol-disulfide exchange reactions, in particular, protein conformational dynamics play an essential role in regulating cysteine reactivity, such as affecting the thiol *pKa* values^3,4,18^. Via combined efforts from both X-ray crystallography and solution NMR spectroscopy, the structures of Trx-coupled ArsC (either *B. subtilis* ArsC *or S. aureus* pI258 ArsC, abbreviated as Bs_ArsC and Sa_ArsC) have been elucidated in various reaction stages, including the transiently formed Trx-ArsC mixed-disulfide complex^15–17,19,20^. However, information concerning the changes of protein dynamics and conformational landscapes along the reaction pathway is still limited, and the underlying mechanism of how each of the five cysteines involved (three from ArsC and two from Trx) become sequentially activated at different reaction steps remains to be further elucidated. We have previously performed the backbone ^15^N NMR relaxation measurements of Bs_ArsC in both the reduced and oxidized states^20^. Herein we use a combination of the Carr-Purcell-Meiboom-Gill (CPMG)-based *R*_2_ relaxation dispersion (CPMG RD) and chemical exchange saturation transfer (CEST) NMR methods^21–24^ to investigate the conformational dynamics of Trx-coupled ArsC during the catalytic cycle, and to gain further understanding of protein thiol-disulfide exchanges. In brief, the CPMG RD method measures the changes of the effective transverse relaxation rates (*R*_2_^*eff*^) at different frequencies ν_CPMG_ (ν_CPMG_ = 1/(4τ_cp_) and τ_cp_ is the delay between the refocusing pulses in the CPMG pulse train), and can be used to extract information such as the exchange rate *k*_*ex*_, the populations of the ground and invisible states *p*_*A*_ and *p*_*B*_, and the absolute value of chemical shift differences |Δω| between the two states^22^. It is sensitive to exchange processes on the 200–2000 s^−1^ regime^24^, but not to motions on slower timescales. The CEST method, which uses the saturation transfer effect to probe the exchanges between a ground state conformation and possible sparsely-populated excited state conformation(s), is complementary to CPMG RD in that it can report on motions on the timescale of ~20–200 s^−1^, and it also has the advantage of being able to directly observe the chemical shifts of the invisible states^21,24^. For clarity, we show a brief summary of the arsenate reduction catalytic cycle in Fig. 1. The ArsC enzyme in different reaction states will be abbreviated as “*re-ArsC*” for the reduced state, “*int-ArsC*” for the C10–C82 disulfide bonded intermediate state, “*ox-ArsC*” for the oxidized state, and “*c-ArsC*” for ArsC in the Trx-complexed state throughout the manuscript. Unless otherwise mentioned, all states of the ArsC sample in the manuscript refer to Bs_ArsC.

## Results

### Sulfate binding of re-ArsC captured by CEST experiments

The *re-ArsC* is the active state of the enzyme with all three essential cysteine residues (C10, C82 and C89) in the free thiol state. It binds the substrate arsenate via the oxyanion-binding P-loop and carries out the initial nucleophilic attack on arsenate using the active C10, forming a covalent C10-HAsO_3_^−^ intermediate (step 1 in Fig. 1). Since the *re-ArsC* structure requires oxyanion binding to become stabilized^19,20,25^, the available solution structure of *re-ArsC*, which was determined in the presence of sulfate, should be regarded as a mimic of the enzyme non-covalently bound with the substrate. To clearly distinguish between the sulfate-bound and free forms of *re-ArsC*, we herein denote the two as *re-ArsC·sulfate* and *re-ArsC*^*free*^, respectively (see Fig. S1 for details of the assignments of *re-ArsC*^*fre*^).

**Figure 2:**
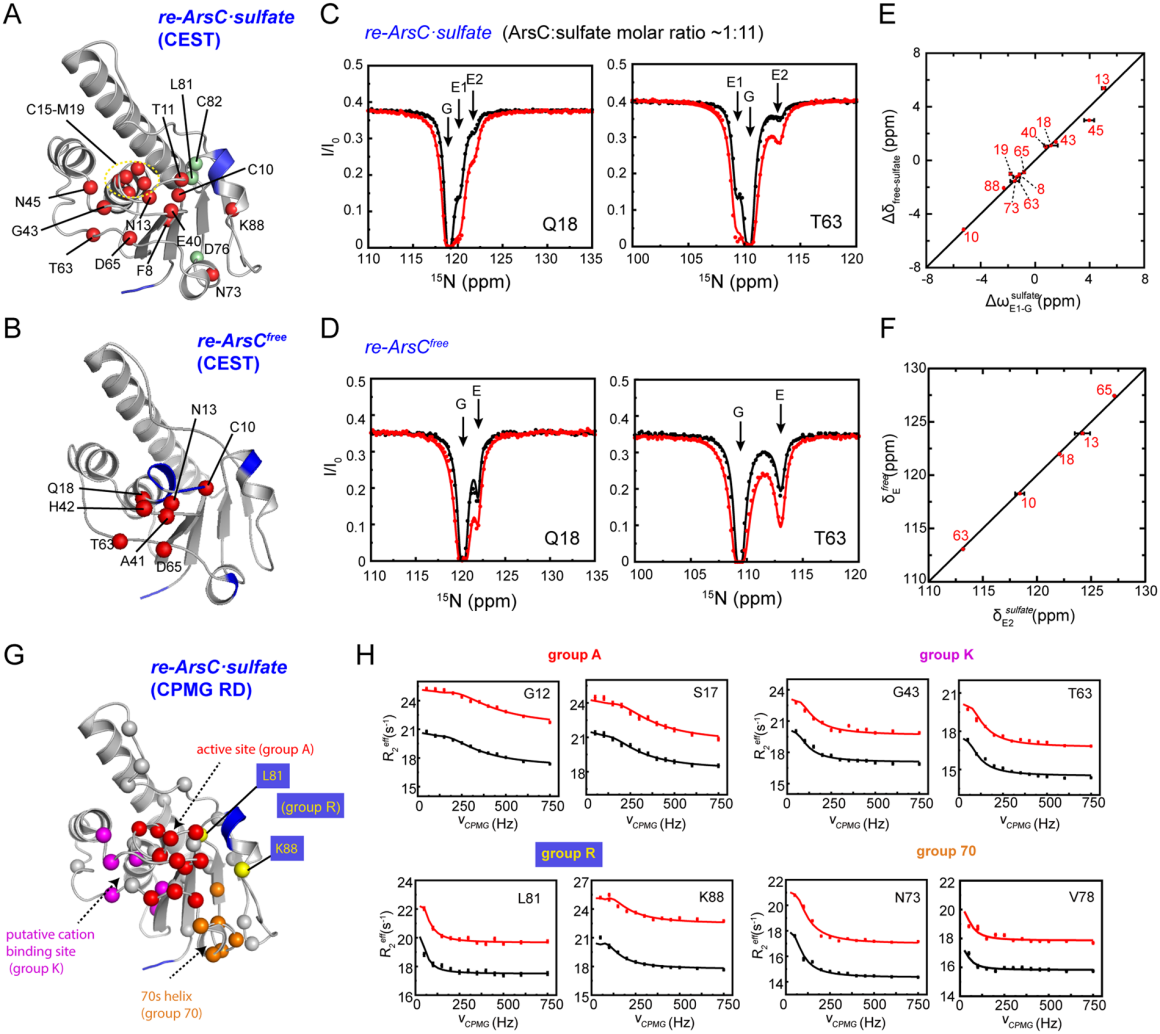
^15^N CEST and CPMG RD results of *re-ArsC*. (**A**,**B**) Mapping of residues showing conformational exchanges in *re-ArsC·sulfate* (**A**) and *re-ArsC*^*free*^ (**B**) identified by CEST experiments onto the structure (shown as spheres). Residues showing sulfate concentration-dependent CEST profiles are shown as red balls in (**A**). Residues with missing backbone amide resonances are colored blue. (**C**,**D**) Representative ^15^N CEST profiles in *re-ArsC·sulfate* (**C**) and *re-ArsC*^*free*^ (**D**) obtained on a 600-MHz spectrometer using B_1_ fields of 8.4 Hz (black) and 13.5 Hz (red). The *re-ArsC·sulfate* sample was prepared with ArsC concentration of 1.8 mM and sulfate concentration of 20 mM. (**E**) Comparison between the Δω values extracted from CEST experiment of *re-ArsC·sulfate* and the chemical shift differences Δδ_free − sulfate_ for residues showing sulfate concentration-dependent CEST profiles. Here Δω_E1-G_^sulfate^ = δ_E1_^sulfate^ − δ_G_^sulfate^, with δ_G_^sulfate^, δ_E1_^sulfate^ corresponding to the chemical shifts of the major and first minor dips in *re-ArsC·sulfate* CEST profiles, respectively, and Δδ_free-sulfate_ = δ_free_ − δ_sulfate_, with δ_free_, δ_sulfate_ corresponding to the chemical shifts in *re-ArsC*^*free*^ and *re-ArsC*·*sulfate*, respectively. Residue numbers are denoted. (**F**) Comparison between the chemical shift of the E2 state in *re-ArsC·sulfate* (δ_E2_^sulfate^) and E state in *re-ArsC*^*free*^ (δ_E_^free^), with residue numbers denoted. (**G**) Mapping of residues showing conformational exchanges in the CPMG RD experiments in *re-ArsC·sulfate* onto the structure (shown as spheres). Residues that are included in the “A”, “K”, “R” and “70” groups are shown in red, magenta, yellow and orange, respectively. Residues with missing backbone amide resonances are colored blue. (**H**) Representative RD profiles of the four groups obtained on 600-MHz (black) and 800-MHz (red) spectrometers.

The ^15^N CEST experiment was carried out on both *re-ArsC·sulfate* and *re-ArsC*^*free*^. In *re-ArsC·sulfate*, a total of 19 non-overlapping residues were identified to undergo exchanges by the CEST experiments, including the segment F8-M19 covering the P-loop region and a number of residues in the adjacent 40 s and 60 s loops, as well as in the 80 s segment (Fig. 2A,B). The depths of the minor dips (corresponding to the excited state) in the CEST profiles are dependent on the sulfate concentrations for all residues in the P-loop and for many residues in neighboring regions (Fig. S2), suggesting that the exchange is probably related to the binding and releasing of the sulfate ion. This scenario is further supported by the high similarity of the Δδ_free-sulfate_ (chemical shifts differences between *re-ArsC*^*free*^ and *re-ArsC·sulfate*) and Δω values (chemical shift differences between the major and minor dips in the CEST profiles) for the majority of residues (Fig. 2E).

In addition, a number of residues in *re-ArsC·sulfate* (including C10, T11, A15-Q18, T63, D65, N73, C82 and K88) harbor at least two distinct minor dips in the CEST profile (Figs 2C and S3A). Analyses using different three-state exchange models^26^ favor a G ⇌ E1 ⇌ E2 exchange processes (G is the ground state, E1 and E2 are two different excited states corresponding to the larger and smaller minor dips) (Fig. S4). The observation that the depths of the third dip are also affected by sulfate concentrations (Fig. S2) further supports this exchange model rather than E1 ⇌ G ⇌ E2 or G ⇌ E2 ⇌ E1 model. Since the G ⇌ E1 exchange reflects the sulfate binding and releasing event, it is rational to assume that E1 corresponds to *re-ArsC*^*free*^. Thus, the E1 ⇌ E2 exchange process observed in *re-ArsC·sulfate* is expected to also exist in *re-ArsC*^*free*^. As expected, six residues (N13, Q18, A41, H42, T63 and D65) show two-state G ⇌ E exchanges in *re-ArsC*^*free*^ (Figs 2D and S3B), and four (N13, Q18, T63 and D65) of these show three-state G ⇌ E1 ⇌ E2 exchanges in *re-ArsC·sulfate*. Furthermore, residue C10 is observed to undergo three-state exchanges in *re-ArsC*^*free*^ and even more complex exchanges in *re-ArsC·sulfate* (Fig. S3A,B). The chemical shifts of the E state in *re-ArsC*^*free*^ and the E2 state in *re-ArsC·sulfate* are essentially similar (Fig. 2F). Taken together, the CEST data reveal two exchange processes around the active site, one corresponding to the sulfate binding with *k*_*ex*_ of ~120 s^−1^, and the other reflecting conformational exchanges between an empty substrate binding pocket with an excited state with *k*_*ex*_ of ~300 s^−1^, where the exchange rates were estimated from the global fitting of residues R16-Q18, G43, T63 and D65 in *re-ArsC·sulfate* using the G ⇌ E1 ⇌ E2 three-state exchange model (Table S1 and Fig. S3C). Moreover, the dissociation constant *K*_*d*_ for sulfate binding to *re-ArsC* is estimated to be ~1 mM based on the CEST results, which is confirmed by isothermal titration calorimetry (ITC) measurements with an estimated *K*_*d*_ of 0.7 mM.

### Multiple conformational exchanges in re-ArsC

To further characterize the motions of *re-ArsC·sulfate*, we employed the CPMG RD method and identified about forty residues undergoing conformational exchanges in *re-ArsC·sulfate* (Figs 2G,H and S5). These residues cluster in four regions surrounding the active site pocket: the P-loop, the 80 s segment, the 40 s and 60 s loops, and the 70 s helix, which is consistent with our previous ^15^N relaxation measurements^20^.

Data analysis indicates that residues in different structural regions are involved in separate local motions (Table 1). A subgroup of 12 residues (including T11-N13, C15-S17, G38-E40, and D65-I67) located on a continuous surface around the active site pocket can be described by a two-state exchange model with global exchange parameters *k*_*ex*_ = 870 ± 58 s^−1^ and *p*_*E*_ = 0.52 ± 0.01%. These residues are designated as group A for clarity (“A” for “active site”). A second group of residues (Q18, V34, S36, G43, N45 and T63) undergo a local exchange process with *k*_*ex*_ = 328 ± 36 s^−1^ and *p*_*E*_ = 1.28 ± 0.09%. These residues locate around a putative potassium binding pocket that was identified in *S. aureus* pI258 ArsC but appears to be inactive in *B. subtilis* ArsC^27,28^, and are designated as group K (“K” for potassium). Residues in the 80 s redox functional segment also exhibit motions different from group A residues. Three residues (G83, D84, A85) are missing in the ^1^H-^15^N HSQC spectrum, indicative of conformational exchanges on the intermediate timescale, whereas both residues L81 and K88 (which we designate as group “R” for “redox”) are estimated to show exchange rates of ~40 s^−1^, which is much slower compared to group A and falls outside the time regime that could be reliably characterized by the RD method. Note that the relationship between *k*_*ex*_ and |Δω| determines whether the conformational exchange falls into the fast exchange (when *k*_*ex*_ > |Δω|) or intermediate exchange (when *k*_*ex*_ ≈ |Δω|) regime on the NMR timescale^29^. Since the |Δω| values are distinct among different residues, the above-mentioned residues in the 80 s segment may still be involved in the same collective motion (with a same global *k*_*ex*_) while displaying different |Δω| values. The ones with |Δω| close to *k*_*ex*_ would be significantly broadened and undetectable. Furthermore, residues D68, D70, L72-N74 and V78 around the 70 s helix (designated as group 70) can be fitted using a two-state exchange model with exchange parameters *k*_*ex*_ = 269 ± 48 s^−1^ and *p*_*E*_ = 1.9 ± 0.2%.

**Table 1:** Summary of the kinetic parameters of ArsC from CPMG RD data.

*re-ArsC·sulfate*			
**Group A**	residues 11–13, 15–17, 38–40, 65–67		
k_ex_ = 870 ± 58 s^−1^	p_E_ = 0.52 ± 0.01%	k_GE_ = 4.5 ± 0.1 s^−1^	k_EG_ = 866 ± 58 s^−1^
**Group K**	residues 18, 34, 36, 43, 45, 63		
k_ex_ = 328 ± 36 s^−1^	p_E_ = 1.28 ± 0.09%	k_GE_ = 4.2 ± 0.1 s^−1^	k_EG_ = 324 ± 36 s^−1^
**Group R**	residues 81, 88		
k_ex_ = 40 ± 20 s^−1^	p_E_ = 6.6 ± 3.0%	k_GE_ = 2.6 ± 0.7 s^−1^	k_EG_ = 37 ± 20 s^−1^
**Group 70**	residues 68, 70, 72–74, 78		
k_ex_ = 269 ± 48 s^−1^	p_E_ = 1.9 ± 0.2%	k_GE_ = 5.1 ± 0.2 s^−1^	k_EG_ = 264 ± 48 s^−1^
***int-ArsC***			
**Conformation-1**	residues 7-9, 20, 22, 24, 34, 35, 50, 62, 68-1, 73, 74, 78, 79-1, 85, 96, 98-1, 102, 104		
k_ex_ = 685 ± 24 s^−1^	p_E_ = 4.9 ± 0.3%	k_GE_ = 33.6 ± 0.1 s^−1^	k_EG_ = 651 ± 24 s^−1^
**Conformation-2**	residues 7-9, 20, 22, 24, 34, 35, 50, 62, 68-2, 73, 74, 78, 79-2, 85, 96, 98-2, 99-2, 102, 104		
k_ex_ = 681 ± 23 s^−1^	p_E_ = 4.9 ± 0.3%	k_GE_ = 33.4 ± 0.1 s^−1^	k_EG_ = 648 ± 24 s^−1^
***c-ArsC***			
	residues 10, 12, 13, 15–18, 20, 36, 39,45, 65, 67, 80, 82, 83, 87–89, 95–97, 100–102		
k_ex_ = 443 ± 22 s^−1^	p_E_ = 2.8 ± 0.1%	k_GE_ = 12.4 ± 0.1 s^−1^	k_EG_ = 431 ± 22 s^−1^

A number of residues located around the P-loop region are identified to exhibit conformational exchanges in *re-ArsC·sulfate* by both CEST and CPMG RD methods, and a subset of these display three-state exchanges in the CEST profile. Comparison of the exchange parameters including *k*_*ex*_, *p*_*E*_ and |Δω| derived from the two methods suggests that for group A residues forming the active site pocket, the CPMG RD and CEST experiments report on different exchange processes. For group K residues (Q18, G43 and T63), although the *k*_*ex*_ value derived from CPMG RD data is close to the *k*_*ex*,2_ value (the apparent exchange rate between E1 and E2 in the three-state exchange model), we did not find apparent correlations between the corresponding chemical shift differences. On the other hand, the CEST profiles of many residues in the P-loop region as well as the 70 s and 80 s segments could not be well fitted globally, therefore prohibiting accurate determination of the exchange parameters, which also indicates that the motions in *re-ArsC·sulfate* may be more complex (Table S1 and Fig. S3). Taken together, *re-ArsC·sulfate* displays highly complex dynamics involving multiple conformational exchange processes for different structural regions. In particular, the active P-loop undergoes at least three different exchange processes, namely the binding of sulfate and exchanges with two different excited states on different timescales.

### Structural characterization of int-ArsC

**Figure 3:**
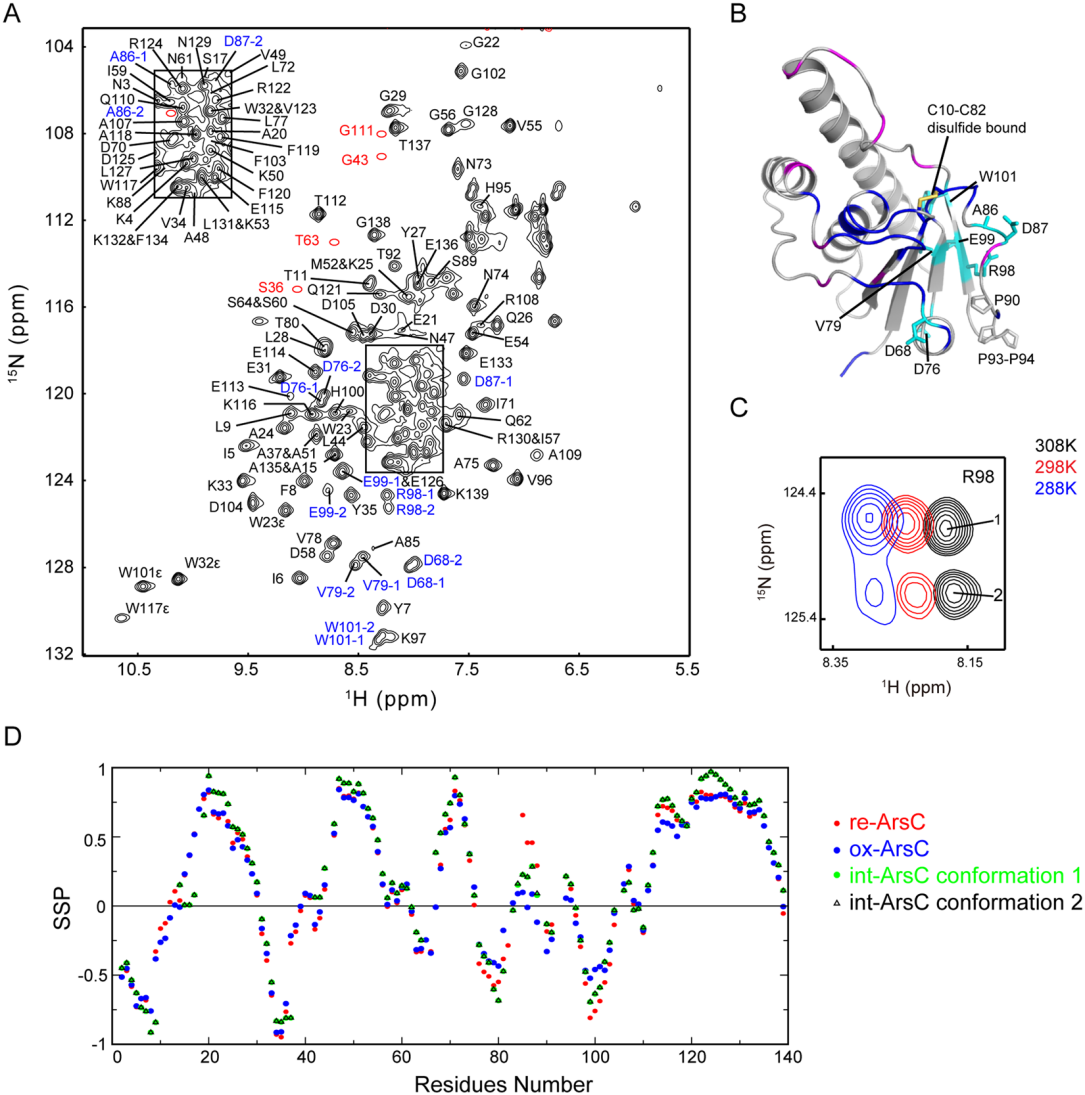
Assignments and structural characterization of *int-ArsC*. (**A**) The ^1^H-^15^N HSQC spectrum of *int-ArsC* labeled with backbone resonance assignments. The positions of the weak resonances are indicated by red circles in the spectrum. Residues showing two sets of peaks are labeled in blue with ‘−1’ and ‘−2’ designating two conformations. (**B**) Mapping of residues with backbone amide resonances missing (blue) or significantly broadened (magenta), and residues with two sets of backbone amide resonances (shown as cyan sticks) onto the structure model of *int-ArsC*. The C10–C82 disulfide bond and the three proline residues in this region are also shown. (**C**) Representative local spectral region showing the two sets of signals from R98 at different temperatures. (**D**) Secondary structure propensity derived from the chemical shifts from ^15^N, ^13^C^α^, ^13^C^β^, ^13^CO, ^1^H^α^ and ^1^H^N^ atoms using the program package SSP^30^ for *re-ArsC*, *ox-ArsC* and *int-ArsC* with the two sets of conformations. SSP values close to 1 indicate high propensity of α-helices, whereas SSP values close to −1 indicate high propensity of β-strands.

The C10–C82 disulfide-bonded *int-ArsC* is an important but short-lived reaction intermediate during the intra-molecular disulfide cascade, and could only be trapped by mutating the C89 residue to quench the reaction. To facilitate dynamic studies, we completed the chemical shift assignments of *int-ArsC* and characterized its structural properties (Fig. 3). Twenty-four out of the total of 139 residues are missing in the ^1^H-^15^N HSQC spectra of *int-ArsC*, and quite a few residues around the active site show broadened resonances, suggesting significant conformational exchanges on the intermediate timescale. Furthermore, a number of residues located near the 80 s segment show two sets of peaks in the HSQC spectrum, indicating dual conformations in slow exchange with each other (Figs 3A–C and S6). Judging from the intensities of the two sets of peaks, the two conformations have a population ratio of approximately 1:1 at 25 °C, whereas decreasing the temperature results in the population increase of one set of the peaks (Fig. 3C). We designate the set of peaks that is dominant at lower temperatures as conformation-1, and the other set as conformation-2.

Previous structural studies showed that the 80 s redox functional segment (C82–C89) undergoes a “helix-to-loop” transition coupled to its self-oxidization during the thiol-disulfide cascade^19^, and *int-ArsC* is the reaction intermediate during this transition from the reduced to oxidized state. Inspection of the 3D NOESY spectra of *int-ArsC* identified no characteristic NOEs of helical conformations in the 80 s segment, whereas secondary structure propensity (SSP) analysis using the assigned ^13^C^α^/^13^C^β^/^13^CO/^1^H^α^/^1^H^N^/^15^N chemical shifts^30^ estimated helical-forming propensities for residues A85-K88 in between the predicted values of *re-ArsC·sulfate* (with ~50% of helical propensity) and *ox-ArsC states* (with close to 0 helical propensity) (Fig. 3D). This indicates that the 80 s segment in *int-ArsC* adopts an “intermediate” conformation, or that the chemical shifts reflect an average over different populations with higher and lower helical contents.

### Global collective motions in int-ArsC

**Figure 4:**
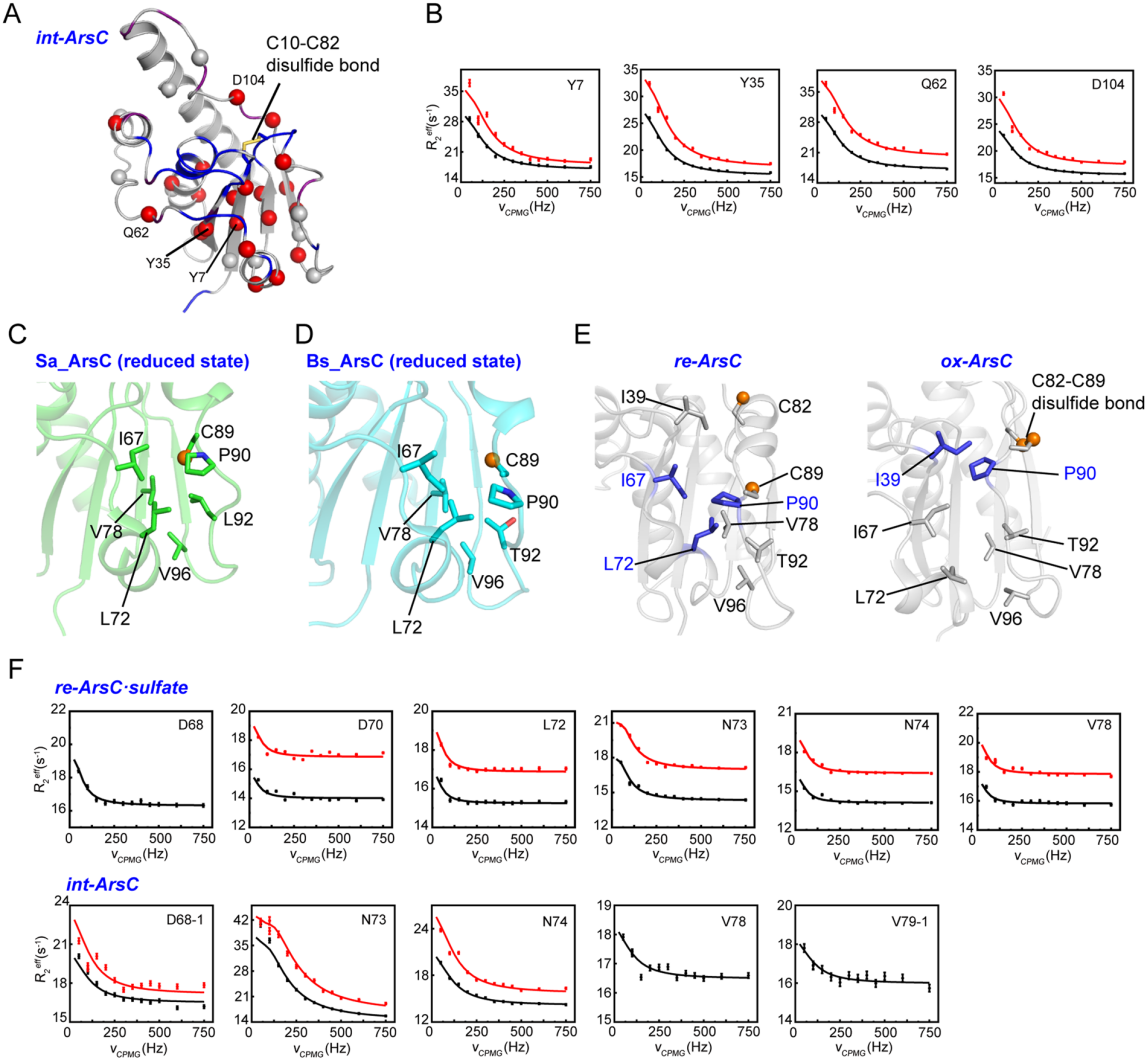
CPMG RD results of *int-ArsC*. (**A**) Mapping of residues showing conformational exchanges in *int-ArsC* identified by CPMG RD experiments onto the structure model (shown as spheres). Residues with missing backbone amide resonances are colored blue, and residues with weak signals are colored purple. (**B**) Representative RD profiles of residues in *int-ArsC* obtained on 600-MHz (black) and 800-MHz (red) spectrometers. (**C**,**D**) The local conformation of the hydrophobic pocket that C89 occupies in the reduced state of Sa_ArsC (**C**, PDB code: 1ljl) and Bs_ArsC (**D**, PDB code: 1z2d). (**E**) Comparison of the local structural changes of the hydrophobic pocket in *re-ArsC* and *ox-ArsC* showing the packing of P90 either with I67, I72 in *re-ArsC* (left) or with I39 in *ox-ArsC* (right). (**F**) Representative RD profiles of residues in the 70 s segment in *re-ArsC·sulfate* (upper panel) and *int-ArsC* (lower panel) obtained on 600-MHz (black) and 800-MHz (red) spectrometers. The data for D68 in *re-ArsC·sulfate* and V78 and V79 in *int-ArsC* obtained at B_0_ field of 800 MHz were excluded from analysis due to relatively large experimental errors.

While the CEST experiment detected no conformational exchanges in *int-ArsC*, the CPMG RD data identified about thirty residues exhibiting millisecond timescale dynamics. These residues spread over different regions of the protein structure, including the central β-sheet and the surrounding α-helices (Fig. 4A,B), reflecting an extremely dynamic and transient nature of this intermediate state. Data analysis revealed that the RD profiles of a total of 21 residues could be described using a global two-state exchange process. These residues are located around the active site pocket and extend to nearly all the regular secondary structural elements, including all strands in the central β-sheet, indicating a global collective motion involving different structural regions.

For residues showing two sets of peaks, both conformation-1 and 2 show similar relaxation dispersion phenomenon and the estimated exchange parameters are essentially similar when using the data set from either conformation. The global exchange parameters for the 21 residues are estimated to be *k*_*ex*_ = 685 ± 24 s^−1^, *p*_*E*_ = 4.9 ± 0.3% when using the data from conformation-1, and *k*_*ex*_ = 681 ± 23 s^−1^, *p*_*E*_ = 4.9 ± 0.3% when using the data from conformation-2. The extracted absolute shift differences |Δω| for residues with two sets of peaks are different from the ^15^N chemical shift differences |Δδ| between the conformations, which is expected since the RD experiment is sensitive to exchanges on fast timescales whereas the existence of double peaks reflects exchanges on slower timescales.

### C10–C82 disulfide bond induces faster motions in the 70s helix

In the previous crystallographic study of Sa_ArsC in the C10–C82 disulfide-bonded intermediate state (*int-ArsC*), Messens and co-workers pointed out that C89 needs to come out of a hydrophobic pocket and move over a distance of ~10 Å to carry out the nucleophilic attack on C82^19^. In the reduced state (*re-ArsC*), this local hydrophobic pocket is formed by several hydrophobic residues in the 70 s segment (I67, L72 and V78 in Sa_ArsC) and the 90 s loop (P90, L92 and V96 in Sa_ArsC) (Fig. 4C). In Bs_ArsC, the corresponding residues are I67, L72, V78, P90, T92 and V96 (Fig. 4D). Though T92 in Bs_ArsC is a polar residue, it uses its methyl group to contact the hydrophobic core, while its hydroxyl group points outward. In the oxidized state (*ox-ArsC*), C89 moves out of the hydrophobic pocket, accompanied by changes of the relative positions of the hydrophobic residues that can be viewed as a sliding between two hydrophobic tracks, one formed by residues L72, V78, I67 and I39, and the other by P90, T92, V96 (Fig. 4E). In particular, the P90 side chain forms contacts with both I67 and L72 in *re-ArsC*, whereas it slides toward the active site and interacts with I39 in the *ox-ArsC*, undergoing the largest conformational change among these hydrophobic residues. Notably, the amide signals of both I39 and I67 are missing in the HSQC spectrum of *int-ArsC*, indicative of intermediate conformational exchanges on the NMR timescale, which might be related with the switching of P90 between two conformations, one with P90 docking at the I67 site (resembling *re-ArsC*) and the other docking at the I39 site (resembling *ox-ArsC*). Moreover, the CPMG RD data show that residues D68, N73, N74, V78 and V79 in the 70 s segment undergo conformational exchanges in *int-ArsC*, which may also be related to the local structural rearrangement that could facilitate the movement of P90 and C89.

Intriguingly, millisecond timescale motion for the 70 s helix is also observed in *re-ArsC* (Table 1 and Fig. 4F), but not in either *ox-ArsC* or the Trx-complexed *c-ArsC* (*vide infra*). In *re-ArsC·sulfate*, the residues in group 70 exhibit local motions with *k*_*ex*_ of ~269 s^−1^, and is uncoupled from the motions at the active site (*k*_*ex*_ ~ 870 s^−1^). Under the assumption of a two-state exchange process $$\begin{array}{c}{k}_{GE}\\ G\,\rightleftharpoons {\rm{E}}\\ {k}_{EG}\end{array}$$ (where G and E stand for the ground and the excited states), the apparent exchange rate constant *k*_*ex*_ is the sum of *k*_*GE*_ and *k*_*EG*_, and the rate constant *k*_*GE*_ for the transition from the ground state to excited state can be calculated by *k*_*GE*_ = *k*_*ex*_ * *p*_*E*_ (*p*_*E*_ is the relative population of the excited state). Therefore, the forward transition rate constant *k*_*GE*_ for group 70 in *re-ArsC·sulfate* is 5 s^−1^. In *int-ArsC*, however, the motions of group 70 residues become coupled to the active site, with a global *k*_*ex*_ of ~680 s^−1^ and *p*_*E*_ is ~4.9%. Thus, the rate constant *k*_*GE*_ increases to 33 s^−1^ in *int-ArsC*, which is about seven-fold faster than in *re-ArsC·sulfate*.

Taken together, the data indicate local millisecond dynamics of the 70 s segment pre-exist in the reduced state, whereas formation of the C10–C82 disulfide bond in *int-ArsC* results in acceleration of the motions, as well as coupling of this local dynamics with the active site pocket. The increased rate for the transition to excited state in *int-ArsC* is favorable for the movement of C89 out of the hydrophobic pocket, and the coupling of motions between the 70 s site and the active site may be important for relaying the large-scale movement of C89 with its subsequent nucleophilic attack on the C10–C82 disulfide.

### ox-ArsC shows limited dynamics

The *ox-ArsC* corresponds to the inactivated state of the enzyme after completing the reduction of one molecule of arsenate. Both CEST and CPMG RD data demonstrate that the dynamics in *ox-ArsC* are largely diminished. Only four residues (G12, S14, N45 and T80) show varying *R*_2_^*eff*^ depending on the ν_CPMG_ field in the RD profiles, whereas the observed deviations of *R*_2_^*eff*^ are quite small (Fig. S7A). Individual fitting results indicate that the four residues are not involved in a collective motion and cannot be fitted globally. The relatively large fitting errors also prohibited reliable estimation of the kinetic parameters. Apparently, the P-loop residues in *ox-ArsC* are significantly rigidified on the millisecond timescale, showing little *R*_2_^*eff*^ variations (Fig. S7B). It is also observed that the *R*_2_^*eff*^ values in this loop are systematically higher in the active *re-ArsC·sulfate* state than the inactive *ox-ArsC* state, indicative of conformational dynamics in *re-ArsC* but not *ox-ArsC* (Fig. S7C). Furthermore, the 80 s segment also gains higher rigidity, and the 70 s segment also shows no conformational exchanges in *ox-ArsC*.

### Global collective motions in c-ArsC

**Figure 5:**
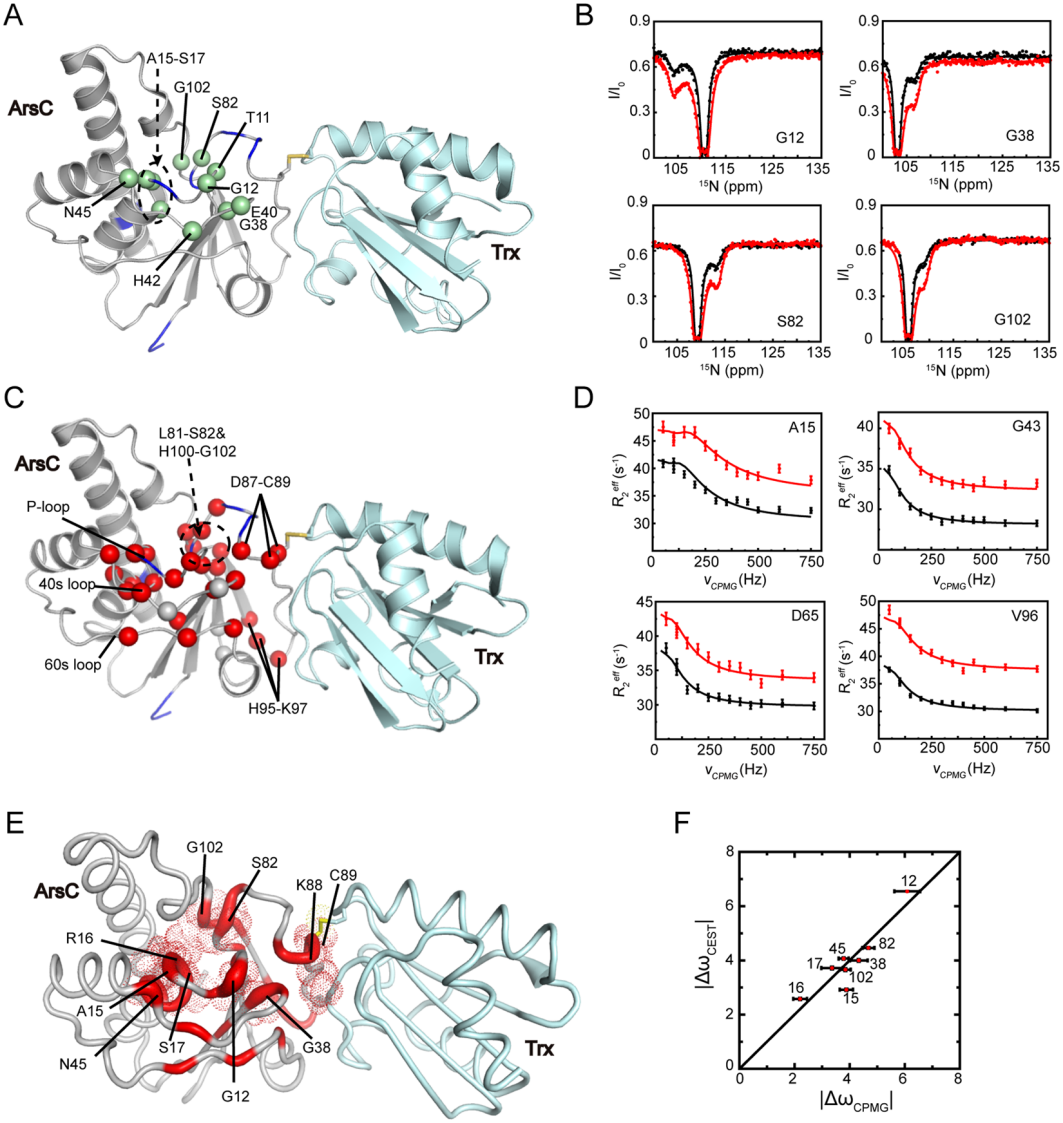
CEST and CPMG RD results of *c-ArsC*. (**A**,**C**) Residues showing conformational exchanges in *c-ArsC* identified by the CEST (**A**) and CPMG RD (**C**) experiments are mapped onto the Trx-ArsC complex structure (PDB ID: 2ipa), shown as green and red spheres, respectively. (**B**) Representative ^15^N CEST profiles of *c-ArsC* obtained on a 600-MHz spectrometer using B_1_ fields of 8.4 Hz (black) and 13.5 Hz (red). (**D**) Representative RD profiles of *c-ArsC* obtained on 600-MHz (black) and 800-MHz (red) spectrometers. (**E**) Mapping of the |Δω| values based on the CPMG RD results onto the *c-ArsC* structure. Residues undergoing collective motions are colored in red, and the thickness of the cartoon tubes reflects the relative values of |Δω|. Residues with |Δω| > 2.0 are shown as dotted spheres. (**F**) Comparison between the absolute Δω values extracted from CEST and CPMG RD experiments for residues in *c-ArsC* with residue numbers denoted.

During the regeneration of ArsC by Trx, a mixed-disulfide Trx-ArsC complex is transiently formed. The CEST method identified a total of eleven residues in *c-ArsC* to undergo conformational exchanges (Fig. 5A,B). These residues are located near the active site pocket and can be analyzed using a two-state exchange model, with a global exchange rate of 440 s^−1^ and a minor state population *p*_*E*_ of ~3.8%. Further, the CPMG RD method also identified about thirty residues to show ν_CPMG_-dependent *R*_2_^*eff*^ values, distributed in a large structural area surrounding the P-loop and the Cys82–Cys89 redox functional segment (Fig. 5C,D). The RD profile for the majority of these residues can be described by a global two-state exchange process, with the estimated exchange parameters of *k*_*ex*_ = 443 ± 22 s^−1^ and minor state *p*_*E*_ = 2.80 ± 0.10% (Table 1). These values are highly similar to the values obtained from the CEST data, suggesting that the two methods may be reporting on the same exchange process. This scenario is further supported by the essential similarity between the absolute chemical shift differences |Δω| extracted from the two methods (Fig. 5F).

The extensive conformational exchanges observed near the active site in *c-ArsC* is quite different from *ox-ArsC*, and more closely resembles *re-ArsC*. Because a stable Trx-ArsC sample needs to be prepared using a C10S/C15A/C82S triple mutant of ArsC and the C89 residue forms an intermolecular disulfide bond with Trx (see Methods), the chemical environment of the residues around the active site pocket are largely affected. Residues exhibiting millisecond dynamics are mostly located within close proximity to the C10, C15, C82 and C89 residues, and therefore a direct comparison of the |Δω| values with the chemical shift differences |Δδ| between *re-ArsC* and *c-ArsC*, or between *ox-ArsC* and *c-ArsC*, is not feasible. Instead, we mapped the |Δω| values onto the *c-ArsC* structure to show the residues that have the largest chemical/conformational differences between the ground and invisible states (Fig. 5E). Residues G12, A15 (mutated from C15), R16, S17 in the P-loop, G38 and N45 in the 40 s loop, S82 (mutated from C82), K88 and C89 in the 80 s segment, and G102 in the 100 s loop show the largest |Δω| values, indicating that they undergo the most significant structural rearrangement in the excited state. The G12, A15-S17, G38, S82 and G102 residues form a circle surrounding the active site pocket, whereas the K88 and C89 residues form the center of the interaction surface with Trx.

### Inter-protein coupling of motions in the Trx-ArsC complex

**Figure 6:**
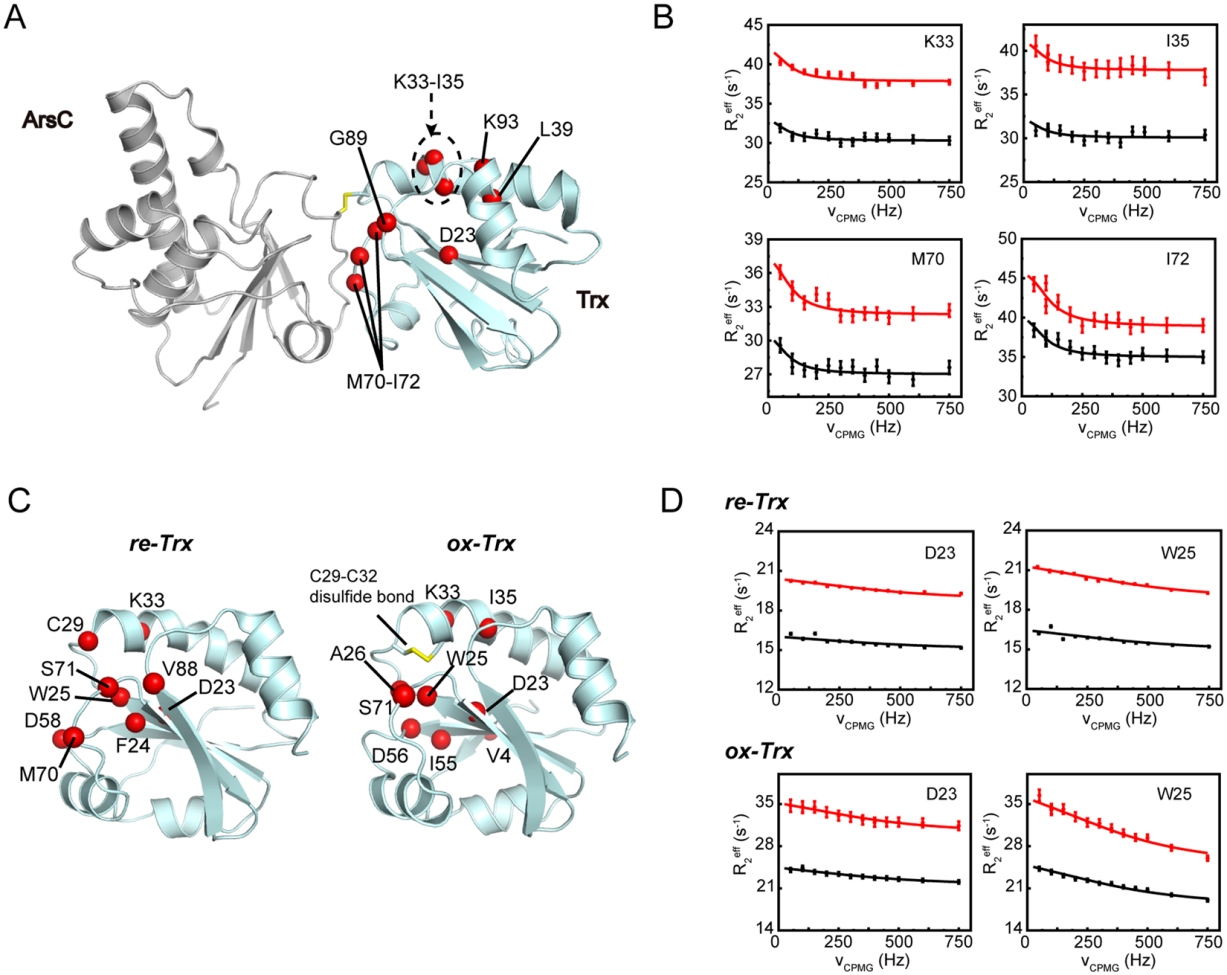
CPMG RD results of Trx in the complex and free states. (**A**,**C**) Residues showing conformational exchanges in *c-Trx* (**A**) or *re-Trx*, *ox-Trx* (B) identified by the CPMG RD experiments are mapped onto the structures (shown as red spheres). (**B**) Representative RD profiles of *c-Trx* (B), *re-Trx* and *ox-Trx* (**D**) obtained on 600-MHz (black) and 800-MHz (red) spectrometers.

The Trx-ArsC complex is transiently formed during the catalytic cycle and a subsequent nucleophilic attack from Trx-C32 quickly resolves the mixed disulfide bond. The global collective motions in *c-ArsC* intrigued us to ask whether the coupling of dynamics is further extended to residues in Trx (designated as *c-Trx*). ^15^N CPMG RD experiments were collected using a Trx-ArsC complex sample with Trx uniformly ^15^N-labeled and ArsC unlabeled. A total of ten residues (D23, K33-I35, L39, M70-I72, G89 and K93) were found to exhibit conformational exchanges in *c-Trx* (Fig. 6A,B). Among these, M70-I72 and G89 locate on the interaction surface that directly contacts ArsC in the complex structure. In particular, M70 side chain plays a main role in complex formation by inserting into a hydrophobic groove on the surface of ArsC^17^. The other residues are clustered around the cysteine residue C32 in Trx and include the acidic D23 which was suggested to play a role in the activation of C32^31–33^. Moreover, the resonance of C29 is weak and that of G30 is missing, indicative of intermediate exchanges.

RD data analysis of *c-Trx* suggests exchange parameters similar to *c-ArsC*, and a combined global fitting using the data from both *c-ArsC* and *c-Trx* estimates the exchange rate constant *k*_*ex*_ = 464 ± 21 s^−1^ and minor state population *p*_*E*_ = 2.70 ± 0.10%, which is essentially identical to the values extracted from the *c-ArsC* data alone. For comparison, we also measured the CPMG RD data of Trx in its reduced and oxidized states (designated as *re-Trx* and *ox-Trx*), both detecting a number of residues exhibiting conformational exchanges around the two catalytic active cysteine residues (Fig. 6C,D). However, the exchange rate constant *k*_*ex*_ is much higher (*k*_*ex*_ ~ 2000–3000 s^−1^) in both *re-Trx* and *ox-Trx* compared to *c-Trx*. The results indicate the formation of the mixed-disulfide complex significantly changes the motions of Trx active site residues and also results in an inter-protein coupling of dynamics.

## Discussion

During the cascade of thiol-disulfide exchange in the arsenate reduction, a total of five catalytic active cysteine residues from two proteins are sequentially activated to perform a series of nucleophilic attacks. It is therefore intriguing to elucidate the structural basis for the regulation of this highly ordered chain of reactions. The CPMG RD and CEST experiments together demonstrate highly complex conformational spaces sampled by ArsC in different reaction stages. In particular, the *re-ArsC·sulfate* shows the most complex dynamics behavior around the active site, with P-loop residues undergoing three or more exchange processes, indicating that the local conformational energy landscape is extremely rugged. In a previous reported kinetic study of ArsC, it was shown that the presence of excess tetrahedral oxyanions such as sulfate is essential for obtaining the optimal enzymatic activity, whereas in the absence of tetrahedral oxyanions the enzyme is unstable and displays low enzymatic activity^25^. Therefore, rather than the apo *re-ArsC*^*free*^ state, a sample with the P-loop stabilized by tetrahedral oxyanions is expected to represent the active state of the enzyme. While the best stabilizing oxyanion is the substrate arsenate itself, it could not be used because it would quickly react with the enzyme to generate the oxidized form. Despite the lower binding affinity (*K*_*d*_ ~ 0.7 mM) to *re-ArsC* compared to the native substrate arsenate (*K*_*m*_ ~ 47 μM for Bs_ArsC)^28^, the sulfate ion has been shown to directly bind the P-loop and act as a good stabilizing agent^20,25,28^. The interaction between sulfate and the P-loop residues could modulate the local dynamics and help shape the active site conformation to gain optimal geometry for the highest catalytic efficiency. In the *re-ArsC·sulfate* state, the P-loop harboring the catalytic essential C10 residue undergoes fast internal motions with *k*_*ex*_ of ~870 s^−1^, which may be important for priming the initial nucleophilic attack. On the other hand, the 80 s segment harboring the C82 and C89 residues undergoes slower motions in the *re-ArsC·sulfate* state, and becomes involved in a fast global collective motion with *k*_*ex*_ of ~680 s^−1^ only after forming the C10–C82 disulfide bond in the *int-ArsC* state. The faster motions in the 80 s segment and the related 70 s helix in *int-ArsC* may also be important in facilitating the subsequent nucleophilic attack by C89. In contrast, in the *ox-ArsC* state in which all three catalytic cysteine residues (C10, C82 and C89) are inactivated, the protein conformational dynamics become largely diminished. Taken together, the conformational dynamics of ArsC in different state are highly correlated with the enzymatic reaction as also suggested by previous studies^17,19,20^.

On the other hand, it remains less well understood how the inter-protein disulfide exchanges between Trx and ArsC are regulated by protein dynamics. In particular, among the two cysteines in Bs_Trx, C29 is active in the free reduced state for attacking downstream protein substrates while C32 is inactive. Only upon formation of the mixed-disulfide Trx-substrate complex can C32 become activated. The activation mechanism of C32 and how this cysteine residue resolves the mixed-disulfide complex has been investigated and under debate in the past decades. This process has proven difficult to study primarily due to the transient nature of the mixed-disulfide complexes. Such complexes usually can only be obtained by trapping the reaction using Trx mutants with the second cysteine (C32 in Bs_Trx) mutated and therefore measurements of the biophysical properties (such as *pKa* values) of this resolving cysteine cannot be made directly in the mixed-disulfide complexes. Moreover, the mixed-disulfide Trx-substrate complexes are usually dynamic and are not easy to be crystallized^34^. The solution NMR structure of *B. subtilis* Trx-ArsC complex previously determined in our lab is one of the few high-resolution structure of Trx complexed with a substrate protein^17,35–38^. The structure reveals an extended hydrophobic interaction surface between the two proteins, but could not provide direct answer as to how Trx-C32 becomes activated in this complex. On the other hand, besides the large scarcity of structural information, there has been a lack of information concerning protein dynamics that has been proven essential for enzyme activity.

In a computational study starting from the solution structure of Trx-ArsC complex, Roos and co-workers suggested an intriguing mechanism for C32^Trx^ activation which is affected by the hydrogen bonding and protonation state of C82^ArsC^ ^18^. In brief, the thiol group of C82^ArsC^ in Trx-ArsC complex has a *pKa* value of 7.9 when it is free of hydrogen-bonding interactions, whereas its *pKa* drops to 6.3 and becomes more easily deprotonated when it is hydrogen bonded to the side chain of R16^ArsC^. Further, when C82^ArsC^ is deprotonated, the thiol group of C32^Trx^ has higher possibility of forming hydrogen bonds with either the amide of C29^Trx^ or W28^Trx^, with C32^Trx^Sγ-C29^Trx^N and C32^Trx^Sγ-W28^Trx^N hydrogen-bonds formed during 60% and 23% of the time when C82^ArsC^ is in the deprotonated state, as compared to the significantly smaller fraction of 1.6% and 0.5% when C82^ArsC^ is neutral^18^. Simultaneous formation of both C32^Trx^Sγ-C29^Trx^N and C32^Trx^Sγ-W28^Trx^N hydrogen-bonds could help lowering the *pKa* of C32^Trx^, facilitating its deprotonation and thus activation. Our current study of the Trx-ArsC complex dynamics reveals a global collective motion involving the catalytic essential residues from both *c-ArsC* and *c-Trx*, as well as residues across the complex interface, which provides strong experimental support for the long-range coupling of conformational fluctuations that enables the propagation of structural changes from one protein to the other. In particular, among the residues involved in collective motions in *c-ArsC*, residues G38^ArsC^, I39^ArsC^ and D87^ArsC^ locate between the active site pocket and the protein-protein interface. Residues I67^ArsC^ and K88^ArsC^ form direct hydrophobic contacts *c-Trx*. Likewise, residues M70-I72^Trx^ in *c-Trx* locate in the protein-protein interface, whereas G89^Trx^ locates in between the interface region and the active site region surrounding C29^Trx^ and C32^Trx^. In addition, residue D23^Trx^ that has been previously proposed to activate C32^Trx^ via a bond water molecule^31–33,39^ is also involved in the collective motion.

Furthermore, the *k*_*cat*_ for the reduction of *ox-ArsC* by *re-Tr*x has been determined to be approximately 1.9 s^−1^ under a buffer conditions of 50 mM Tris (pH 8.0), 50 mM K_2_SO_4_ and 0.1 mM EDTA^34^. Our current CEST and CPMG RD data of *c-ArsC* demonstrate a two-state exchange process with the forward rate constant *k*_*GE*_ = 13 s^−1^. Assuming the excited state E resembles the conformation in which C82^ArsC^ becomes hydrogen-bonded with R16^ArsC^, and using the values of 60% and 23% from the computational results as the probability of C32^Trx^ in forming hydrogen bonds with C29^Trx^ or W28^Trx^ in this conformational state, we could estimate the rate constant of C32^Trx^ simultaneously forming two hydrogen bonds with C29^Trx^ and W28^Trx^ to be 13 * 0.6 * 0.23 = 1.8 s^−1^, which is highly similar to the *k*_*cat*_ value. This further suggests that inter-protein coupling of dynamics could indeed play a central role in regulating the activation of cysteine thiol groups during disulfide exchanges, and may represent the rate-limiting step in the case of Trx-ArsC redox reaction.

The coupling of motions among different structural regions is not only observed in the Trx-ArsC complex, but also in the C10–C82 disulfide-bonded *int-ArsC* state. The millisecond timescale motion of the 70 s helix, which is important for activating C89^ArsC^, is coupled to the active site only in this intermediate state but not in the reduced state. C89^ArsC^, as well as C32^Trx^, functions as a resolving cysteine (C_R_) that resolves an unstable disulfide bond (C10–C82 in *int-ArsC*, and C89^ArsC^-C29^Trx^ in the Trx-ArsC complex) in transiently formed, energetically unfavorable reaction intermediates. These C_R_ residues are inactive in the reduced state of the enzyme and becomes rapidly activated in the transiently-formed intermediate. The intra-protein and inter-protein coupling of motions observed in *int-ArsC* and the Trx-ArsC complex may reflect a common scenario of protein dynamics-regulated activation of cysteine thiol groups in thiol-disulfide exchange reactions.

As discussed earlier, the mechanism for protein thiol-disulfide transfer reaction is difficult to study and remains in debate for the past twenty years. Moreover, the transient nature of Trx-substrate mixed-disulfide intermediate makes experimental measurement of the *pKa* of the resolving cysteine unfeasible by current available methods. Our current study highlights both intra- and inter-protein coupling of millisecond motions, strongly suggesting the cysteine thiol activities could be regulated by long-range propagation of enzyme dynamics. The results are also highly supportive of the working model of Trx-catalyzed thiol-disulfide exchange reaction as proposed by Roos *et al*. by computational methods. The advance NMR methods in detecting the sparsely populated excited states and extracting the kinetic parameters for the exchange processes shed new light on understanding thiol-disulfide exchange reactions, and when further combined with more detailed computational investigations, would hopefully provide a more comprehensive understanding in this field.

## Methods

### Sample Preparations

Protein expression, labeling and purification of *B. subtilis* ArsC and Trx proteins (including mutants) were similar to previously reported^17,20^. The C10–C82 disulfide-bonded *int-ArsC* sample was prepared using the ArsC_C15AC89S mutant via incubation with 0.3% H_2_O_2_ at room temperature for 1 h followed by gel filtration chromatography. The protein concentration was kept below 1 mg/ml during the incubation to minimize intermolecular disulfide bond formation. Formation of the C10–C82 disulfide bond was verified by Ellman’s test^40^ using 5, 5′-dithio-bis 2-nitrobenzoic acid (DTNB) as the reagent, which reacts with free thiols to yield TNB^2−^ and can be monitored by absorption at 412 nm. The Trx-ArsC mixed-disulfide complex was prepared using the Trx_C32S and ArsC_C10SC15AC82S mutants and following the previously reported 5,5-dithiobis(2-nitrobenzoic acid) incubation protocol^17,34^. The Trx-ArsC complex samples were prepared with ^15^N-labeled ArsC and unlabeled Trx or ^15^N-labeled Trx and unlabeled ArsC. The purities of the protein samples were determined to be greater than 95% as judged by SDS-PAGE. The NMR samples of *re-ArsC*·*sulfate*, *ox-ArsC* and Trx-ArsC complexes were prepared in a buffer containing 20 mM Tris-HCl (pH 6.85), 40 mM KCl, 20 mM urea and 20 mM Na_2_SO_4_, the samples of *re-ArsC*^*free*^ and *int-ArsC* were prepared in a buffer containing 20 mM Tris-HCl (pH 6.85), 40 mM KCl, 20 mM urea and 40 mM NaCl, and the samples of re-Trx and ox-Trx were prepared in a buffer containing 20 mM Tris-HCl (pH 6.85). Excess dithiothreitol (DTT) was added in the samples of the re-ArsC and re-Trx to ensure a reducing environment. D_2_O was added to 5% for field lock and 2,2-dimethyl-2-silapen-tanesulfonic acid was used as the internal chemical shift reference.

### Backbone resonance assignments of re-ArsC^free^ and int-ArsC

The NMR experiments for re-ArsC^*free*^ and *int-ArsC* backbone assignments were carried out at 25 °C on Bruker Avance 500-MHz and 600-MHz spectrometers equipped with four RF channels and cryogenic triple resonance probes with pulsed field gradients. The backbone chemical shift assignments were obtained by using the conventional three-dimensional HNCA, HNCACB, HNCO, CBCA(CO)NH experiments^41^. All NMR spectra were processed using NMRPipe^42^ and analyzed using NMRView^43^.

### Backbone ^15^N CPMG RD measurements

^15^N-labeled ArsC samples were prepared with protein concentrations of 0.7 mM and were argon-flushed. The ^15^N CPMG RD experiments^44^ were carried out at 25 °C on Bruker Avance 600- and 800-MHz spectrometers equipped with cryogenic probes. A constant transverse relaxation time of T_CPMG_ = 60 ms and 80 ms were used for ArsC and Trx, respectively, whereas T_CPMG_ = 40 ms was used for the Trx-ArsC complex. Data were recorded for fourteen different ν_CPMG_ values of 50, 100 (x2), 150, 200, 250, 300, 350, 400, 450, 500, 600 and 750 Hz. Here ν_CPMG_ = 1/(4τ_cp_), where τ_cp_ is the delay between refocusing pulses during the CPMG pulse train. All data were recorded at three different ^15^N carrier frequencies to circumvent off-resonance effect and to obtain reliable relaxation dispersion profiles for signals located in different regions of the spectrum. The spectra were processed using NMRPipe^42^ and the peak intensities were measured using NMRView^43^. Residues exhibiting conformational exchanges on appropriate timescales would show a dispersion profile of *R*_2_^*eff*^ values dependent on ν_CPMG_. The effective transverse relaxation rates *R*_2_^*eff*^ were determined using the equation *R*_*2*_^*eff*^ = (−1/T_CPMG_)ln(*I*_*νCPMG*_/*I*_0_), where T_CPMG_ is the constant transverse relaxation time, *I*_0_ is the intensity measured in the reference spectrum, and *I*_*νCPMG*_ is the intensity measured at different CPMG field strengths ν_CPMG_^45^. Uncertainties in *R*_2_^*eff*^ were calculated as Δ*R*_2_^*eff*^ = (1/T_CPMG_)(Δ*I*/*I*_*νCPMG*_), where Δ*I* is the average standard deviation of peak intensities estimated from repeat measurements^46^. By using the cpmg_fit software from L. Kay and D. Korzhnev^47^, all dispersion data were fitted to the Richard-Carver equation^48^ assuming a two-state exchange model without assumption regarding the exchange regime. Fitting uncertainties were extracted using the covariance matrix method^49^. The dispersion curves for individual residues were generated using the GLOVE^50^ program by fixing the *k*_*ex*_, *p*_*G*_*p*_*E*_ and Δ*ω* parameters. For global data fitting, clustering of residues were based on comparisons of the estimated *k*_*ex*_, *p*_*E*_ parameters obtained from individual fittings, while taking into account of their locations in the three-dimensional protein structure. In addition, for residues showing three-state exchanges in the CEST data, we also tried using three-state exchange models to fit the CPMG data but did not improve the results.

### Backbone ^15^N CEST experiments

^15^N-labeled ArsC samples were prepared with protein concentrations of 1.8 mM and were argon-flushed. ^15^N CEST experiments^21^ were carried out at 25 °C on a Bruker Avance 600-MHz spectrometer. A total of 191 2D data sets were acquired with the ^15^N carrier frequencies positioned from 100 ppm to 138 ppm at a spacing of 0.2 ppm (12.16 Hz) during the irradiation time of *T*_*EX*_ = 800 ms. In all experiments, irradiation field strengths B_1_ of 8.4 ± 0.2 Hz and 13.5 ± 0.2 Hz were used, and a 2.7 kHz field ^1^H decoupling composite pulse sequence (90_x_-240_y_-90_x_) was applied during the *T*_*EX*_ period. Data without using the B_1_ field during the *T*_*EX*_ period was recorded as the reference experiment. B_1_ calibration was carried out following the previously reported methods^51^. All the data sets were processed using the NMRPipe program^42^, and peak intensities were obtained by NMRView^43^. The CEST profiles for the individual residues were generated by calculating the intensity ratios *I/I*_0_ versus the varied ^15^N carrier frequencies, where *I*_0_ is the intensity measured in the reference spectrum, and *I* is the intensity measured with the application of the B_1_ field. The uncertainties of peak intensities were estimated from repeat measurements. For residues showing only one minor dip, the data were fitted to a two-state exchange model using the python program ChemEx (https://github.com/gbouvignies/chemex) as described previously^21^, and the fitting uncertainties were extracted using the covariance matrix method^49^. Unlike *c-ArsC*, the *re-ArsC·sulfate* state displays highly complex dynamics and many residues show two or more minor dips. For these residues, the data were individually fitted using the three-state exchange models with an in-house written Matlab script from B. Yu and D. Yang^26^. Briefly, the CEST data were fitted using three different three-state models (model 1: G ⇌ E1 ⇌ E2; model 2: G ⇌ E2 ⇌ E1; model 3: E1 ⇌ G ⇌ E2), and the G ⇌ E1 ⇌ E2 model was found to best describe the profiles (Fig. S4). The detailed equations for fitting the data using three-state models were described in ref.^26^, and the fitting uncertainties were extracted by calculating the inverse of the Jacobian matrix^52^. Furthermore, ^15^N CEST experiments were collected using protein samples at 0.5 mM and 2 mM concentrations by employing a residue-selective 1D CEST pulse scheme^53^ to exclude the possibility that the observed CEST effects were due to protein oligomerization at high concentrations (Fig. S8).

## Electronic supplementary material

Supplementary Information

## Data Availability

The assigned chemical shifts of *re-ArsC*^*free*^ and *int-ArsC* have been deposited in the BioMagResBank (http://www.bmrb.wisc.edu/) under the accession number of 27283 and 27329.
