# Supplementary material for: Intra- and inter-protein couplings of backbone motions underlie protein thiol-disulfide exchange cascade
Source: Sci Rep. 2018 Oct 18;8:15448. doi: 10.1038/s41598-018-33766-4 (PMC6193951; doi:10.1038/s41598-018-33766-4)
Supplement: Supplementary file 1 — Supplementary Information [file 41598_2018_33766_MOESM1_ESM.pdf]

Supplemental Information for "Intra- and inter-protein couplings of backbone motions underlie protein thiol-disulfide exchange cascade"

Wenbo Zhang[1,3], Xiaogang Niu[2,3], Jienv Ding[1,3,#], Yunfei Hu[2,3,*], Changwen Jin[1,2,3,4,*]

* To whom correspondence should be addressed: Yunfei Hu, E-mail: yunfei@pku.edu.cn; Changwen Jin, E-mail: changwen@pku.edu.cn.

The supporting information includes Table S1 and Figures S1-S7.

Table S1: Three-state global fitting[1] results of the ^{15}N CEST data of *re-ArsC·sulfate*

$k_{ex,1}$ (s^{-1})	$k_{ex,2}$ (s^{-1})		p_{E1} (%)	p_{E2} (%)
123 ± 12	298 ± 46		5.8 ± 0.2	1.1 ± 0.1
Residue No.	$\Delta\omega_{E1,G}$ (ppm)	$\Delta\omega_{E2,E1}$ (ppm)	$\Delta\omega_{E2,G}$ (ppm)	$\lvert\Delta\omega\rvert_{CPMG}$ (ppm)[2]
16	-2.61 ± 0.04	-4.32 ± 0.10	-6.93 ± 0.03	1.74 ± 0.26
17	2.66 ± 0.02	2.78 ± 0.09	5.44 ± 0.03	3.90 ± 0.36
18	1.15 ± 0.01	1.77 ± 0.04	2.92 ± 0.02	1.59 ± 0.10
43	1.53 ± 0.08	-0.29 ± 0.35	1.24 ± 1.21	1.56 ± 0.11
63	-1.35 ± 0.02	3.95 ± 0.02	2.60 ± 0.02	1.49 ± 0.07
65	-0.84 ± 0.03	-3.00 ± 0.05	-3.84 ± 0.04	2.13 ± 0.13

[1] Due to the significantly increased number of unknown parameters in three-state exchange model, the kinetic parameters were estimated from global fitting to prevent over-fitting. The CEST data were fitted to a G$\rightleftharpoons$E1$\rightleftharpoons$E2 model where $k_{ex,1}$ and $k_{ex,2}$ correspond to exchange rates between G$\rightleftharpoons$E1 and E1$\rightleftharpoons$E2, respectively. The CEST data of a total of six residues were used in the global fitting. The data of residues C10, T11, N13 and C15 in the P-loop were excluded from the global fitting due to large fitting errors or abnormal values. Notably, C10 and T11 show more than two minor peaks, suggesting even more complicated dynamics. The CEST profiles of the other residues also could not be well fitted globally using the three-state exchange model. In these cases, only the chemical shift information ($\Delta\omega$ value) could be extracted with high fidelity.

[2] The $\lvert\Delta\omega\rvert$ values extracted from the CPMG RD experiments are listed for comparison.

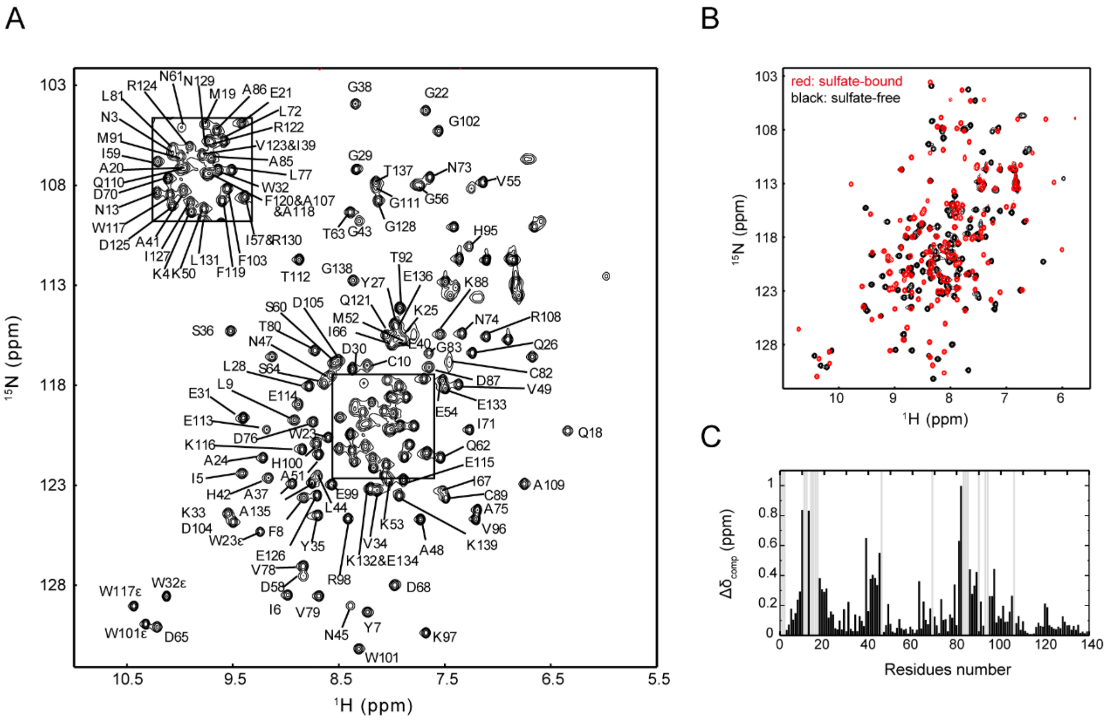

Fig. S1: Spectral comparison of *re-ArsC* in the presence or absence of sulfate. (A) The ^{1}H-^{15}N HSQC spectrum of *re-ArsC^{free}* labeled with backbone resonance assignments. (B) An overlay of the HSQC spectra of *re-ArsC^{free}* (black) and *re-ArsC·sulfate* (red). (C) The composite chemical shift differences between *re-ArsC^{free}* and *re-ArsC·sulfate* calculated using the empirical equation $\Delta\delta_{comp=}\sqrt{\Delta\delta_H{}^2 + (\Delta\delta_N/6.5)^2}$, where $\Delta\delta_H$ and $\Delta\delta_N$ are the chemical shift differences of ^{1}H and ^{15}N nuclei, respectively. Grey bars indicate the residues that are unobservable in at least one of the spectra.

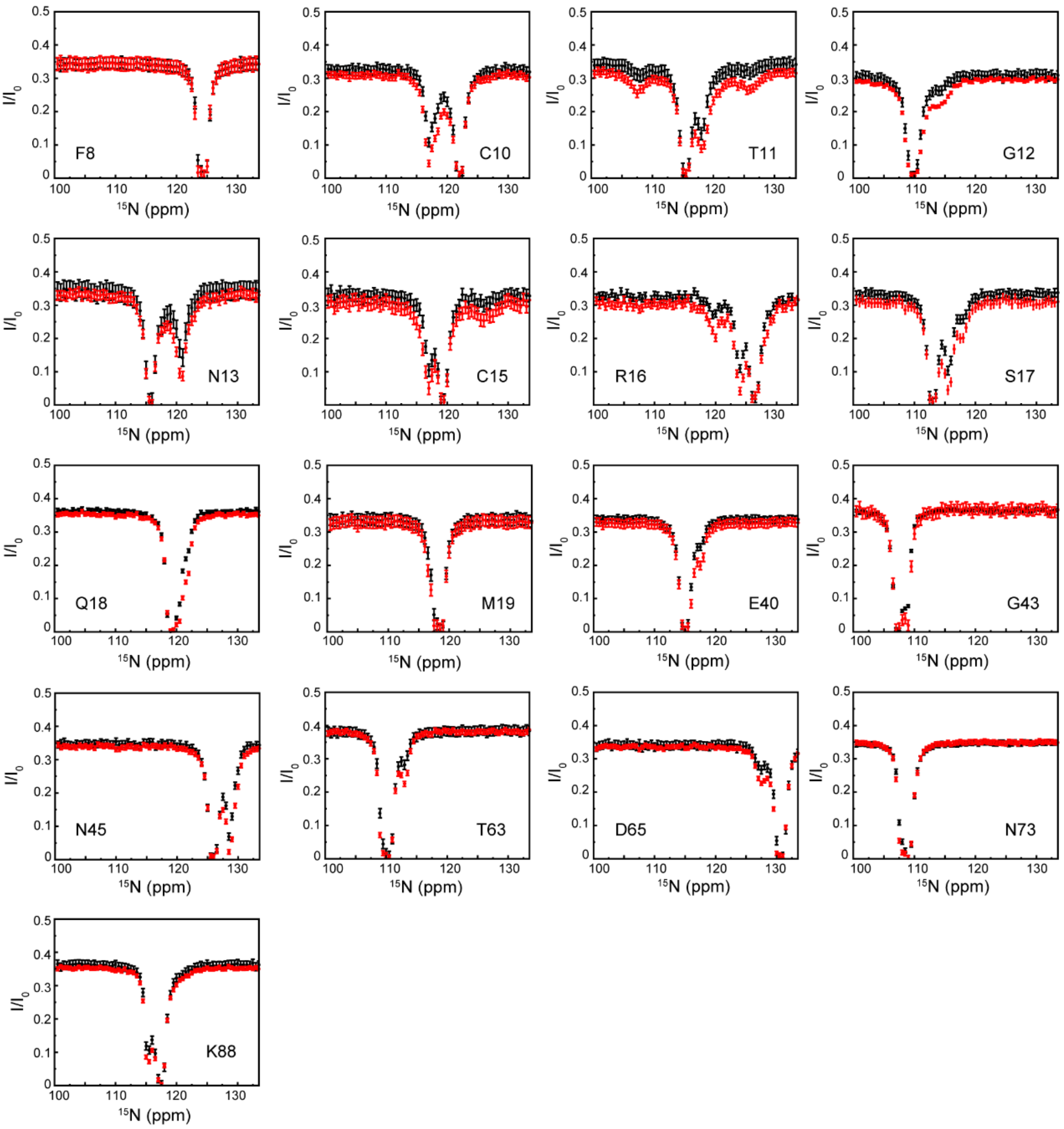

Fig. S2: ^{15}N CEST profiles of residues in *re-ArsC·sulfate* dependent on the concentration of sulfate. The data were obtained on a 600-MHz spectrometer using B_1 field of 13.5 Hz under the protein concentration of 1.5 mM and protein:sulfate molar ratios of 1:5 (red) and 1:10 (black). The data were acquired with ^{15}N carrier frequencies positioned from 100 ppm to 138 ppm at a spacing of 0.5 ppm during the irradiation time of T_{EX}= 800 ms. The minor dip deepens at lower sulfate concentration (red profiles).

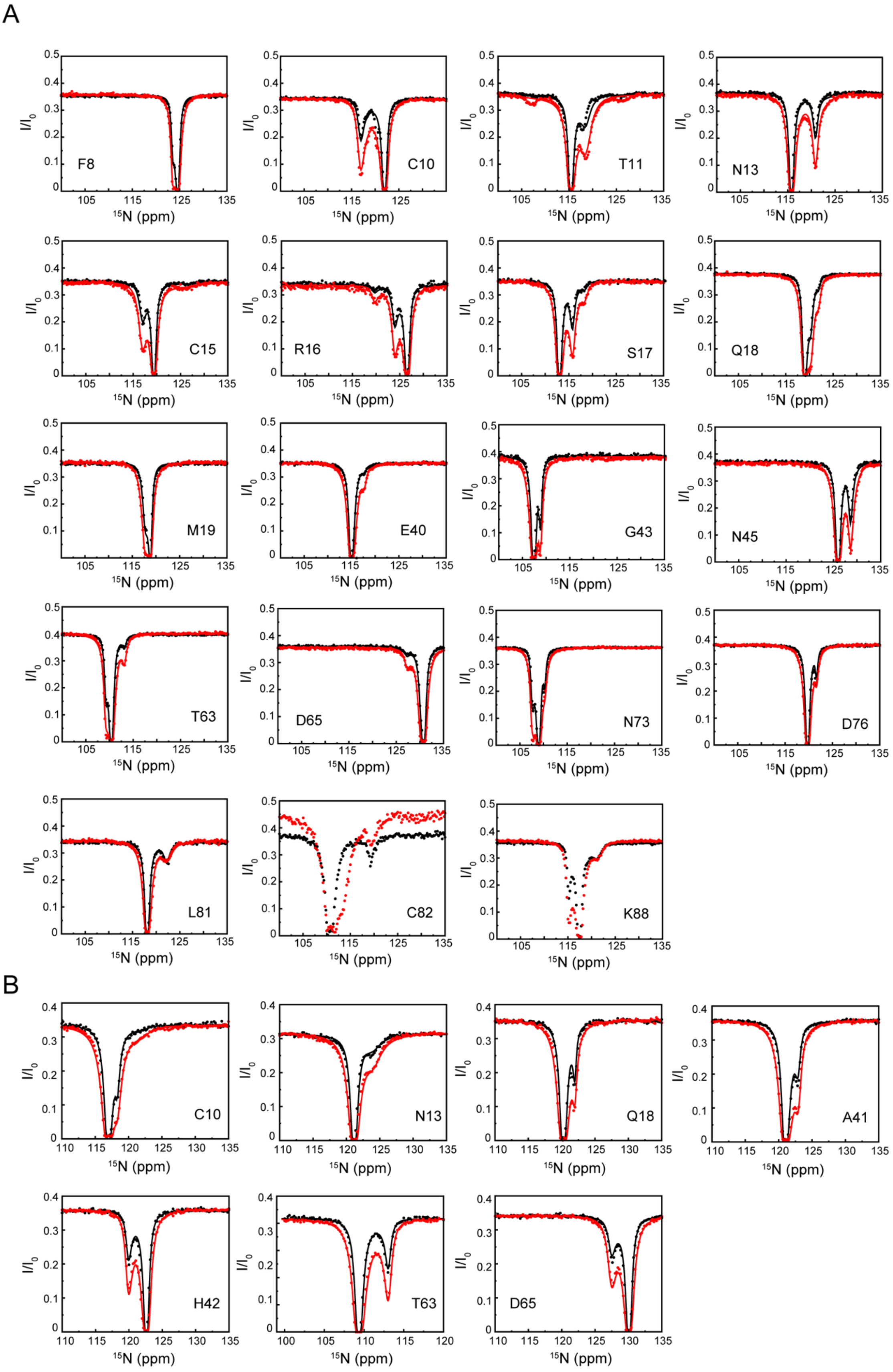
A
F8
C10
T11
N13
C15
R16
S17
Q18
M19
E40
G43
N45
T63
D65
N73
D76
L81
C82
K88
B
C10
N13
Q18
A41
H42
T63
D65
I/I0
15N (ppm)

C

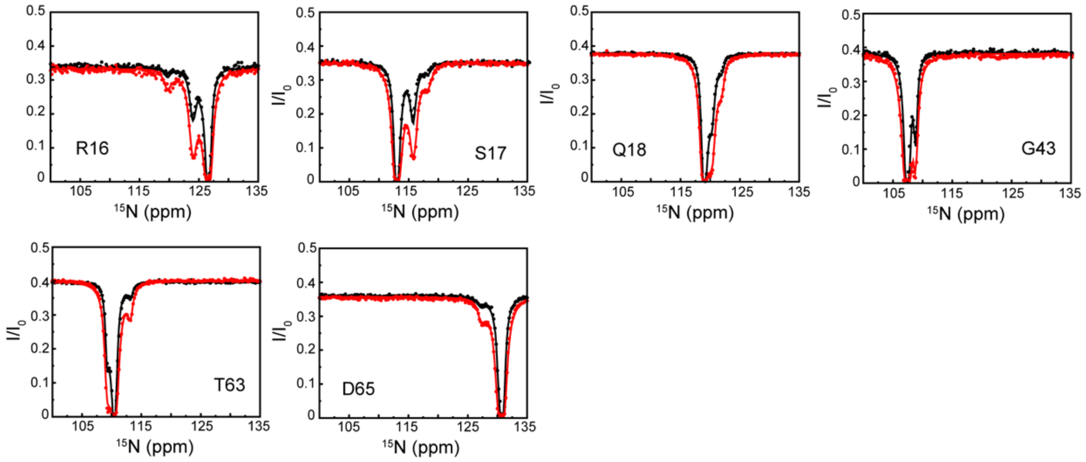

Fig. S3: ^{15}N CEST profiles of residues in *re-ArsC·sulfate* (A,C) and *re-ArsC^{free}* (B). (A) The CEST profiles of *re-ArsC·sulfate* with F8, M19, E40, N45, D76 and L81 individually fitted to a two-state exchange model and other residues individually fitted to a three-state exchange model (G⇌E1⇌E2). The CEST profiles of residue C82 in *re-ArsC·sulfate* are significantly broadened, whereas the data of residue K88 could not be well fitted using either two-state or three-state exchange models, and therefore only the original data are shown. The amide signals of A41 and H42 overlap with other resonances and are thus excluded from analysis. (B) The CEST profiles of *re-ArsC^{free}* with C10 individually fitted to a three-state exchange model (G⇌E1⇌E2) and other residues individually fitted to a two-state exchange model. The amide signals of residues T11, C15, R16, S17 are missing, those of E40 and L81 overlap with other peaks, and the CEST profiles of F8, M19, G43, N45, N73, D76, C82, K88 show only one apparent major dip (or it is possible that the minor peak is too small or too close to the major dip to be observed), and are thus excluded from analysis. (C) Global fitting of the CEST profiles of residues R16-Q18, G43, T63 and D65 in *re-ArsC·sulfate* using a three-state exchange model (G⇌E1⇌E2). All data were obtained on a 600-MHz spectrometer using B_1 fields of 8.4 Hz (black) and 13.5 Hz (red).

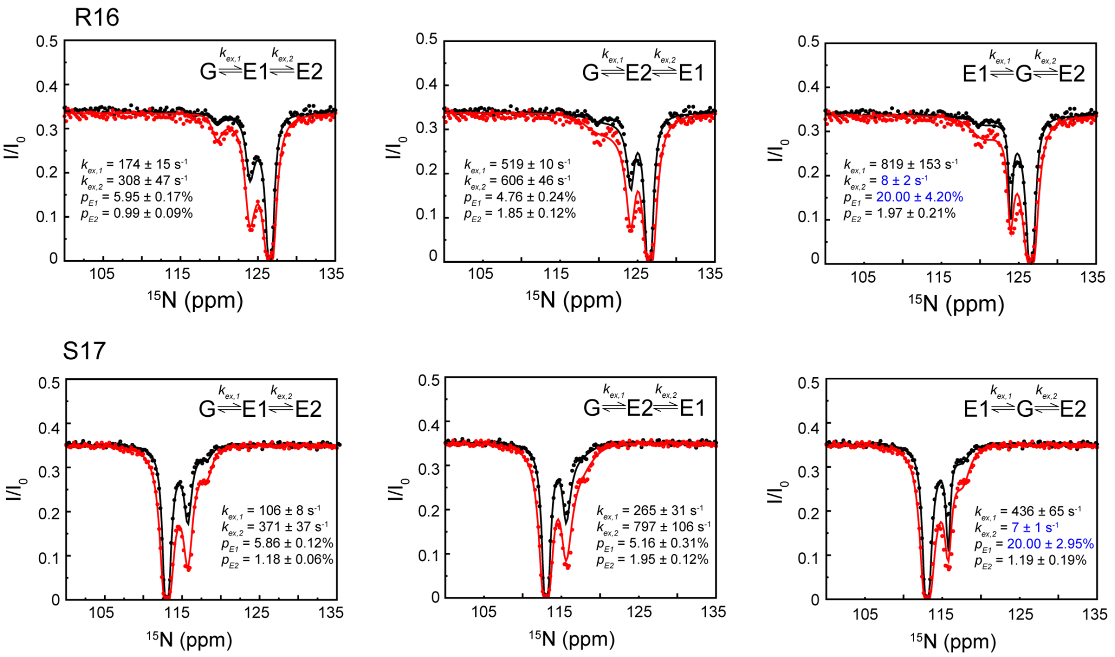

Fig. S4: Model selection for three-state exchange in CEST data analysis. Representative experimental (dots) and calculated CEST profiles (solid lines) using model 1: G⇌E1⇌E2 (left panel), model 2: G⇌E2⇌E1 (middle panel) and model 3: E1⇌G⇌E2 (right panel). The data were obtained on a 600-MHz spectrometer using B_1 fields of 8.4 Hz (black) and 13.5 Hz (red). The $k_{ex,1}$, $k_{ex,2}$, p_{E1} and p_{E2} extracted from the data are annotated and the obvious abnormal values are colored blue.

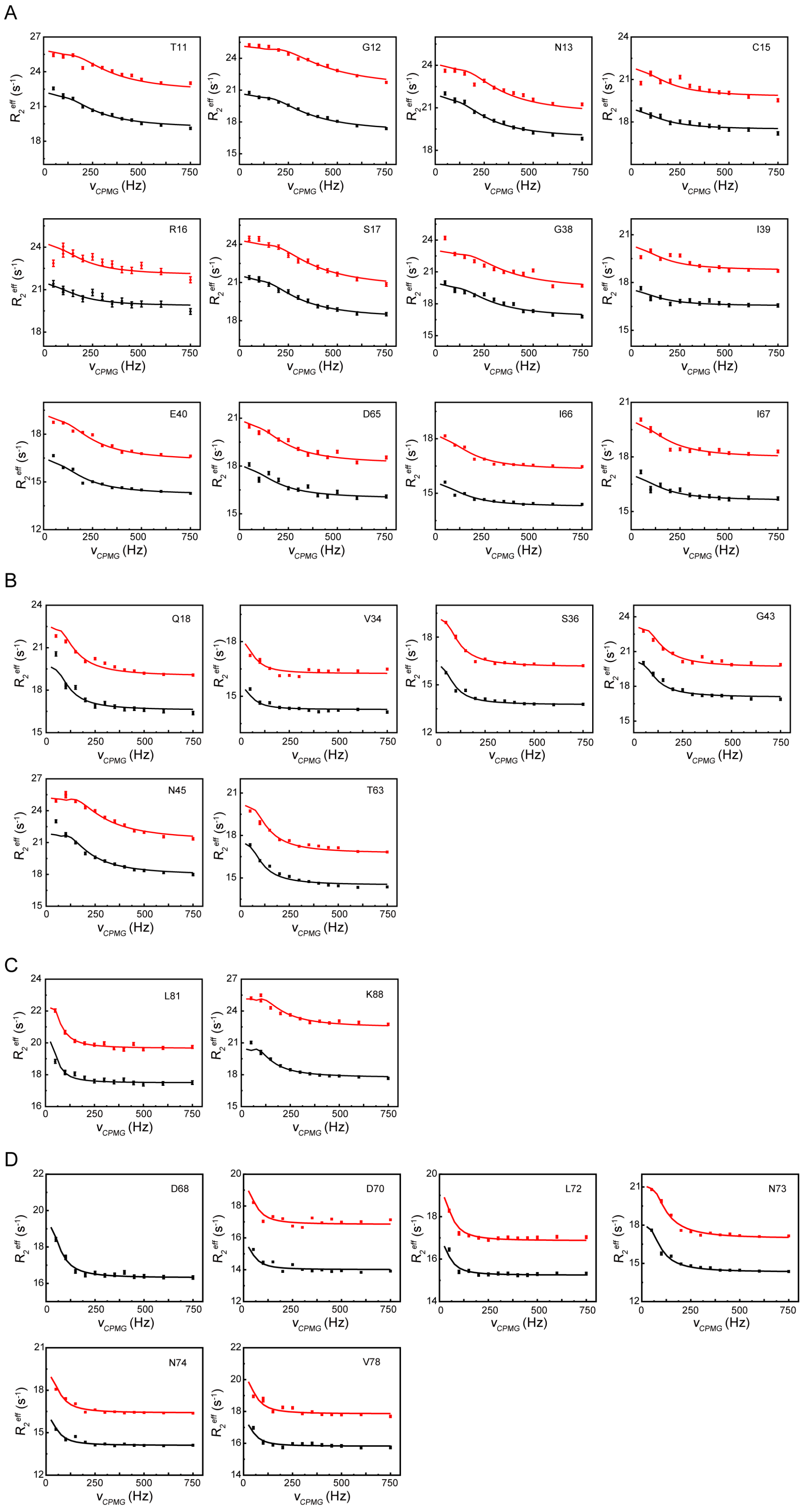

A
T11
G12
N13
C15
R16
S17
G38
I39
E40
D65
I66
I67
B
Q18
V34
S36
G43
N45
T63
C
L81
K88
D
D68
D70
L72
N73
N74
V78
R_2^{eff} (s^{-1})
ν_{CPMG} (Hz)

Fig. S5: Global fitting results of the CPMG RD data for different groups of residues in *re-ArsC·sulfate*. (A-C) CPMG RD profiles and global fitting curves of group “A” (A), group “K” (B), group “R” (C) and “group 70” (D) residues in *re-ArsC·sulfate* as described in the manuscript. The relaxation dispersion profiles were obtained at B_0 fields of 800 MHz (red) and 600 MHz (black), and the data were globally fitted to a two-state exchange model. The data for D68 obtained at B_0 field of 800 MHz was excluded from analysis due to relatively large experimental errors.

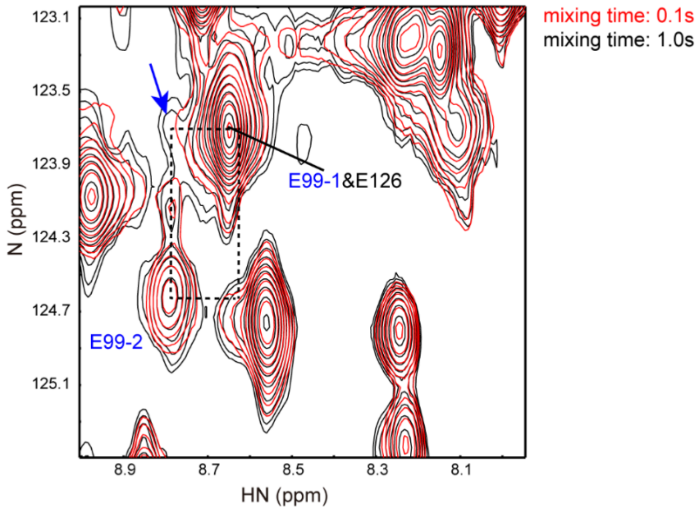

Fig. S6: ZZ-exchange experiment result for residue E99 in *int-ArsC*. E99 shows two sets of peaks and are labeled in blue with '-1' and '-2' designating two conformations. The E99-1 is overlapped with E126. One of the detected cross-peaks originating from the exchange between the two conformations is indicated with blue arrows. The data were obtained at B_0 fields of 700 MHz with mixing time 0.1s (red) and 1s (black). Other residues with dual conformations are located in more crowded spectral regions and their expected cross-peaks in ZZ-exchange experiment cannot be resolved.

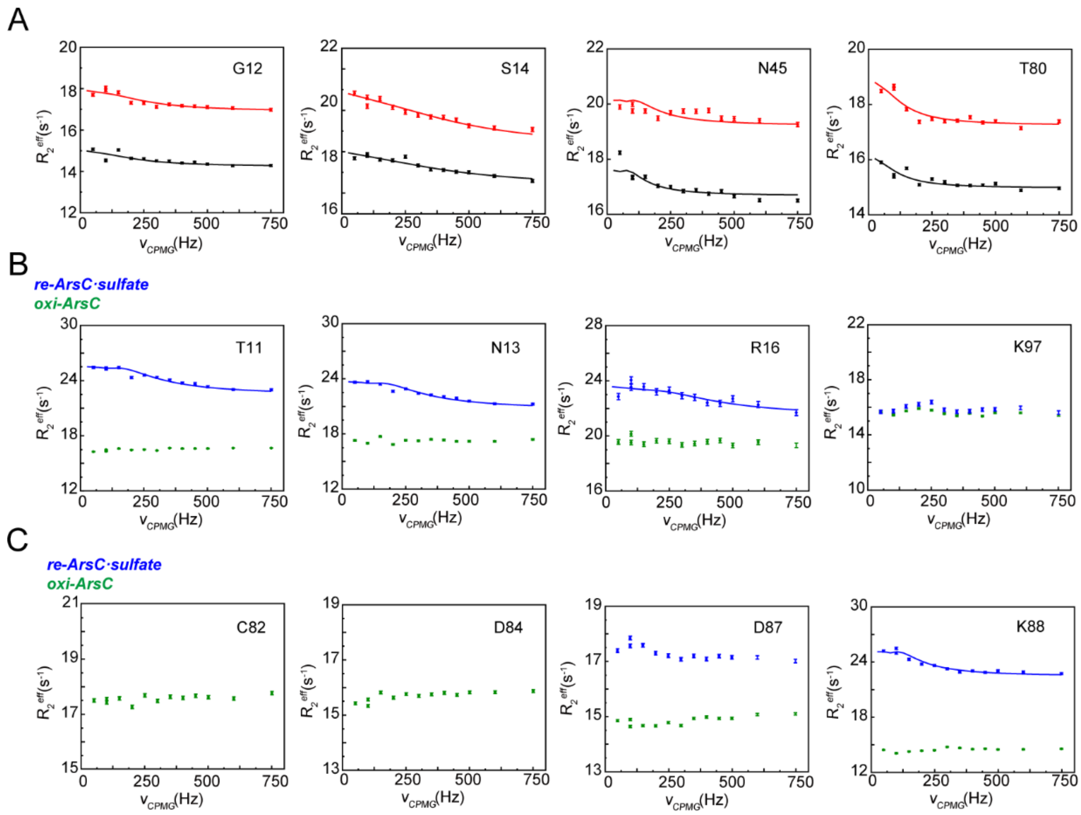

Fig. S7: CPMG RD profiles reveal limited dynamics in *ox-ArsC*. (A) CPMG RD profiles for residues G12, S14, N45 and T80 in *ox-ArsC*. The fitted curves were derived from individual fittings of the data. The relaxation dispersion profiles were obtained at B_0 fields of 800 MHz (red) and 600 MHz (black), and the data were individually fitted to a two-state exchange model. (B-C) Representative comparisons of the RD profiles of residues in the P-loop and the 80s segment in the reduced (blue) and oxidized (green) states obtained on an 800-MHz spectrometer. The profiled of residue K97 which shows no conformational exchanges in either state is shown in the rightmost panel of (B) for comparison. Curves from individual fitting results for residues T11, N13, R16 and K88 are shown, while for other data the fitting procedure failed. The RD profiles of residues C82 and D84 in *re-ArsC* are not shown due to the absence or weakness of signals.

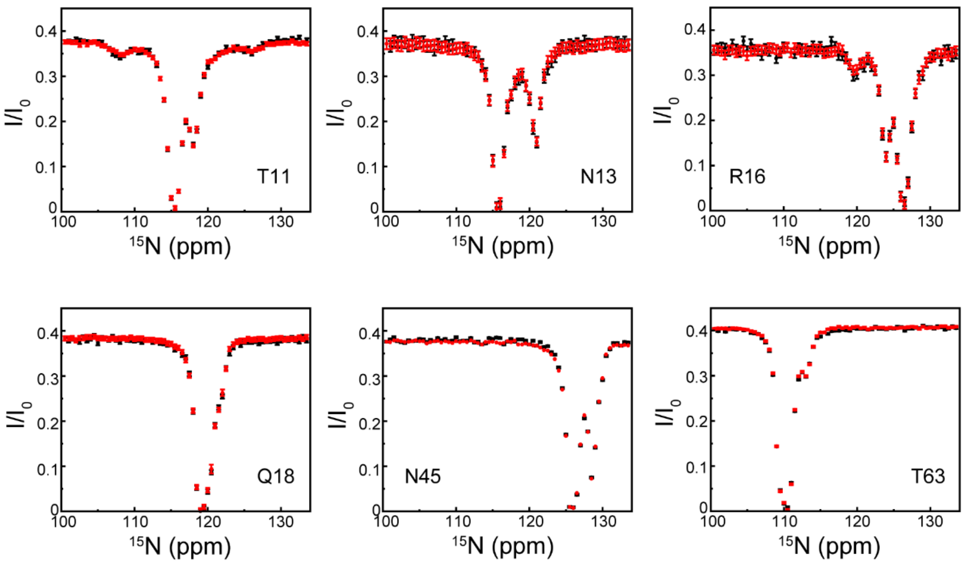

Fig. S8: Comparison of the ^{15}N CEST profiles at different protein concentrations. Representative CEST profiles of *re-ArsC·sulfate* acquired at protein concentrations of 0.5 mM (black) and 2.0 mM (red). The data were obtained on a 600-MHz spectrometer using a B_1 field of 13.5 Hz and irradiation time of 800 ms by employing a residue-selective 1D CEST pulse scheme.
